# Collagen-binding IL-12-armoured STEAP1 CAR-T cells reduce toxicity and treat prostate cancer in mouse models

**DOI:** 10.1038/s41551-025-01508-3

**Published:** 2025-10-23

**Authors:** Koichi Sasaki, Vipul Bhatia, Yuta Asano, Jakob Bakhtiari, Pooja Kaur, Chuyi Wang, Takumi Matsuo, Olivier Dubois, Po-Chuan Chiu, Donny Gun, Charanjit Singh, Ioanna Panagi, Laurine Noblecourt, Maria Nikolaidi, Truman Chong, Gerardo Javier, Saul J. Priceman, Aude G. Chapuis, John K. Lee, Jun Ishihara

**Affiliations:** 1 Department of Bioengineering, Imperial College London, London, UK; 2 Human Biology Division, Fred Hutchinson Cancer Center, Seattle, WA USA; 3 Division of Hematology/Oncology, Department of Medicine, David Geffen School of Medicine at UCLA, Los Angeles, CA USA; 4 Translational Science and Therapeutics Division, Fred Hutchinson Cancer Center, Seattle, WA USA; 5 Department of Microbiology, Immunology and Molecular Genetics, UCLA, Duarte, CA USA; 6 Department of Hematology and Hematopoietic Cell Transplantation, City of Hope, Duarte, CA USA; 7 Department of Immuno-Oncology, Beckman Research Institute of City of Hope, Duarte, CA USA; 8 Division of Medical Oncology, University of Washington, Seattle, WA USA; 9 Parker Institute for Cancer Immunotherapy at UCLA, Los Angeles, CA USA; 10 Exploratory Oncology Research and Clinical Trial Center (EPOC), National Cancer Center, Chiba, Japan

**Keywords:** Cancer immunotherapy, Molecular medicine, Gene therapy, Protein delivery, Synthetic biology

## Abstract

Immunosuppressive microenvironments, the lack of immune infiltration, and antigen heterogeneity pose challenges for chimaeric antigen receptor (CAR)-T cell therapies applied to solid tumours. Previously, CAR-T cells were armoured with immunostimulatory molecules, such as interleukin 12 (IL-12), to overcome this issue, but faced high toxicity. Here we show that collagen-binding domain-fused IL-12 (CBD-IL-12) secreted from CAR-T cells to target human six transmembrane epithelial antigen of prostate 1 (STEAP1) is retained within murine prostate tumours. This leads to high intratumoural interferon-γ levels, without hepatotoxicity and infiltration of T cells into non-target organs compared with unmodified IL-12. Both innate and adaptive immune compartments are activated and recognize diverse tumour antigens after CBD-IL-12-armoured CAR-T cell treatment. A combination of CBD-IL-12-armoured CAR-T cells and immune checkpoint inhibitors eradicated large tumours in an established prostate cancer mouse model. In addition, human CBD-IL-12-armoured CAR-T cells showed potent anti-tumour efficacy in a 22Rv1 xenograft while reducing circulating IL-12 levels compared with unmodified IL-12-armoured CAR-T cells. CBD fusion to potent payloads for CAR-T therapy may remove obstacles to their clinical translation towards elimination of solid tumours.

## Abstract (web summary)

CAR-T cells are engineered to express CBD-IL-12 upon STEAP1 recognition to enhance tumour-specific immune responses in mice.

## Main

Chimaeric antigen receptor (CAR)-T cell therapy has demonstrated remarkable efficacy against previously incurable blood cancers^1–3^. However, the therapy has not yet been very effective against solid cancers. The immunosuppressive tumour microenvironment (TME) limits infiltration and activation of CAR-T cells and endogenous anti-tumour immune cells^4^. Antigen heterogeneity in solid tumours poses additional difficulties for CAR-T cells, which are designed to recognize specific antigens to lyse cancer cells^4,5^.

Interleukin 12 (IL-12) is a cytokine that can remodel the immunosuppressive, ‘cold’ TME in multiple cancer types for favourable therapeutic outcomes^6^. IL-12 is a potent inducer of interferon-γ (IFNγ)^6,7^, which promotes trafficking and infiltration of T, natural killer (NK) and NKT cells into tumours through induction of chemokines, such as C-X-C motif chemokine ligand 9 (CXCL9)^8,9^. IL-12 induces proliferation of T and NK cells and enhances their cytotoxic capacity^10^. IFNγ increases major histocompatibility complex (MHC)-I expression^11^ and matures cross-presenting dendritic cells (DCs)^10,12^ to promote cellular immunity against diverse tumour-associated antigens. IL-12 also reduces myeloid-derived suppressor cell (MDSC) numbers by facilitating their maturation to DCs and macrophages^13^.

Despite great promise, the clinical application of IL-12 has been hampered by dose-limiting immune-related adverse events (irAEs)^14,15^, strongly suggesting the need for optimized drug delivery systems. Several studies have attempted to use the ability of tumour-reactive T cells to migrate into tumour tissue for delivery of IL-12 (refs. ^16,17^). The nuclear factor of activated T cells (NFAT)-responsive promoter enables the engineered T cells to express payloads (that is, anti-tumour biologics) upon recognition of tumour antigen, thereby restricting systemic concentration of the payload^18^. NFAT promoter-driven induction of IL-12 was reportedly safer than its constitutive expression^19^. However, in a Phase I trial, 50% of metastatic melanoma patients who received ≥300 million NFAT-IL-12-equipped tumour-infiltrating lymphocytes exhibited grade 3 or 4 hepatotoxicity, which hampered clinical development^20^. Additional technological advances to counteract the toxicity of IL-12 are required to combine IL-12 with CAR-T cells for clinical use.

We previously showed that intravenously injected A3 collagen-binding domain (CBD) of von Willebrand factor (vWF) effectively accumulates in tumours, due to tumour-specific collagen accessibility^21^. A recombinant fusion of CBD to IL-12 (CBD-IL-12) improved both safety and anti-tumour efficacy^22^. Intravenous CBD-IL-12 induced long-lasting elevation of intratumoural IFNγ levels compared with unmodified IL-12. CBD fusion to IL-12 significantly reduced liver damage marker alanine transaminase (ALT) activity and serum IFNγ.

Metastatic castration-resistant prostate cancer (mCRPC) is a largely incurable solid tumour, with a median overall survival of only 3 years^23^. We have recently identified a promising tumour-associated antigen, six transmembrane epithelial antigen of prostate 1 (STEAP1), which is more broadly expressed than prostate-specific membrane antigen in mCRPC^24^. We have developed second-generation CAR-T cells against human STEAP1 (hSTEAP1), which showed significant, yet non-curative efficacy in multiple prostate cancer models in mice^24^. Intravenously injected CBD-IL-12 protein and STEAP1 CAR-T cells synergized to extend the survival of prostate cancer-bearing mice^24^. We recently initiated a first-in-human trial of STEAP1 CAR-T cells for men with mCRPC (NCT06236139).

Here we further engineered STEAP1 CAR-T cells to express CBD-IL-12 upon STEAP1 recognition to enhance tumour-specific immune responses. We hypothesized that fusion of CBD to IL-12 as a cargo would decrease the systemic toxicity associated with armouring T cells with unmodified IL-12.

## Results

### CBD-IL-12-armoured STEAP1 CAR-T cells efficiently kill cancer cells in vitro

**Fig. 1:**
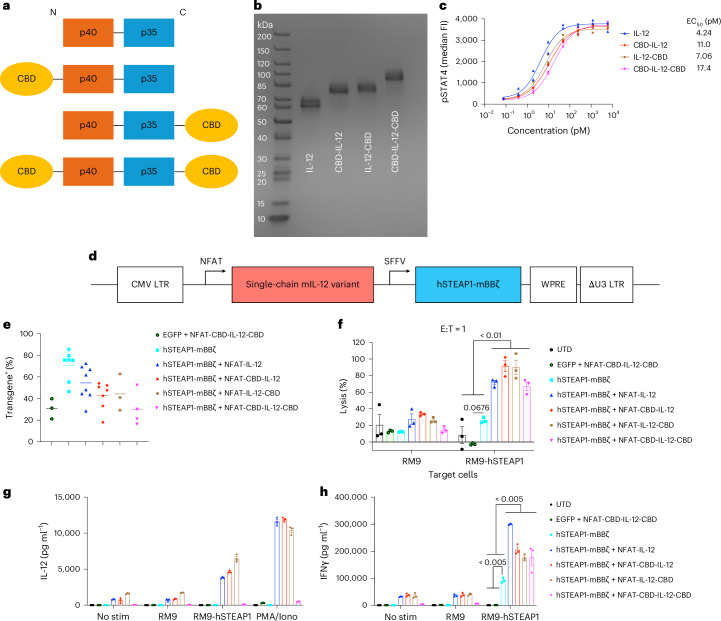
Characterizations of CAR-T cells armoured with a collagen-binding IL-12 in vitro. **a**, Schematic of designs and configurations of single-chain mouse IL-12 variants used in this study. **b**, scIL-12 variants were analysed by SDS–PAGE under reducing conditions with Coomassie blue staining. **c**, Dose–response relationship of phosphorylated STAT4 (pY693) with scIL-12 variants in primary mouse CD8^+^ T cells (*n* = 2 per condition, technical duplicates). EC_50_, half-maximal effective concentration. **d**, Schematic of self-inactivating gamma-retroviral vectors used in this study. **e**, Primary mouse T cells transduced with the indicated gamma-retroviral vectors were assessed for the expression of transgenes by flow cytometry (biological replicates with mean). **f**, RM9-hSTEAP1 cells or unmodified RM9 cells labelled with calcein-AM were co-cultured with CAR-T cells at an effector/target ratio of 1:1 for 24 h (*n* = 3 technical replicates, mean ± s.e.m.). **g**,**h**, Primary mouse CAR-T cells (50,000 CAR-T cells per well) were left untreated (No stim), stimulated with RM9 cells, RM9-hSTEAP1 cells or PMA/Iono for 24 h. **g**, Secreted IL-12 variants and **h**, IFNγ were quantified by ELISA (*n* = 3 technical replicates, mean ± s.e.m.). Statistical analyses were performed using one-way analysis of variance (ANOVA) with Tukey’s test (**f**,**h**) (within RM9-hSTEAP1 co-culture samples). *P* values are shown in figures, and detailed *P* values for Fig. 1f,h are provided in the Source data file. Source data

**Extended Data Fig. 1:**
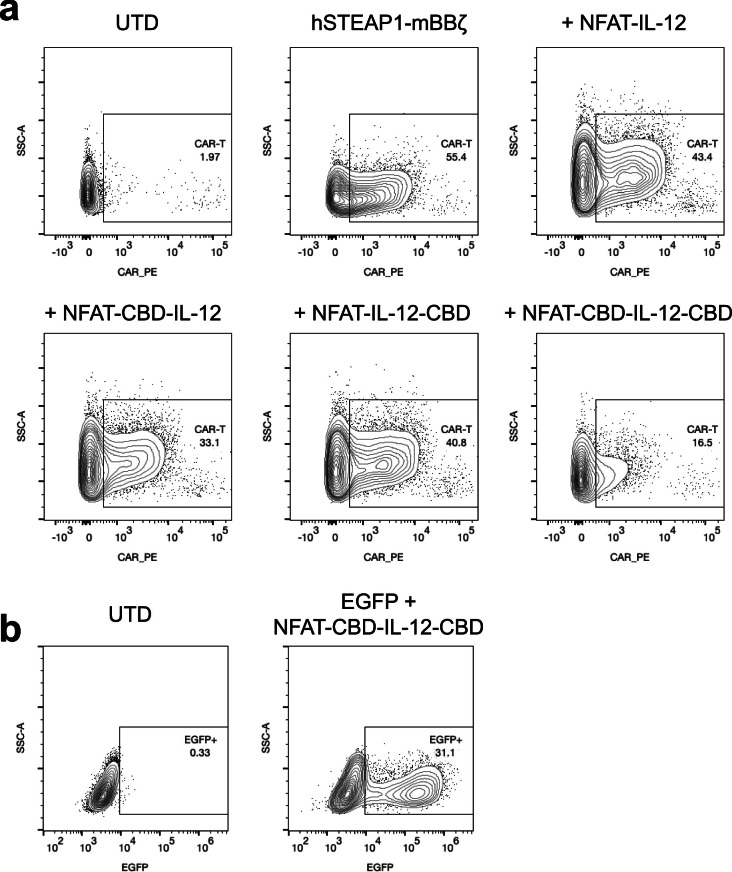
Transduction efficiency of the gamma-retroviral vectors for mouse T cells. Primary mouse T cells were analyzed 4 days after transduction. (**a, b**) Representative flow cytometric contour plots showing (**a**) CAR or (**b**) EGFP expression, related to Fig. 1e.

We designed single-chain (sc) mouse IL-12 variants with different positions of CBD fusion, all of which showed potent half-maximal effective concentration (EC_50_) values (Fig. 1a–c and Supplementary Table 1). We next designed gamma-retroviral vectors for constitutive expression of the mouse CAR targeting hSTEAP1 (ref. ^24^) and NFAT-driven expression of an IL-12 variant (Fig. 1d). Mouse primary T cells were transduced with IL-12 variant-armoured CAR constructs; we noted some reduction in transduction efficiency in proportion to the length of the transgenes (Fig. 1e and Extended Data Fig. 1a). A vector for constitutive expression of enhanced green fluorescent protein (EGFP) and NFAT-driven expression of CBD-IL-12-CBD (CBD on both ends) was also prepared as a control to test IL-12 production triggered by CAR–antigen interaction (Extended Data Fig. 1b).

**Extended Data Fig. 2:**
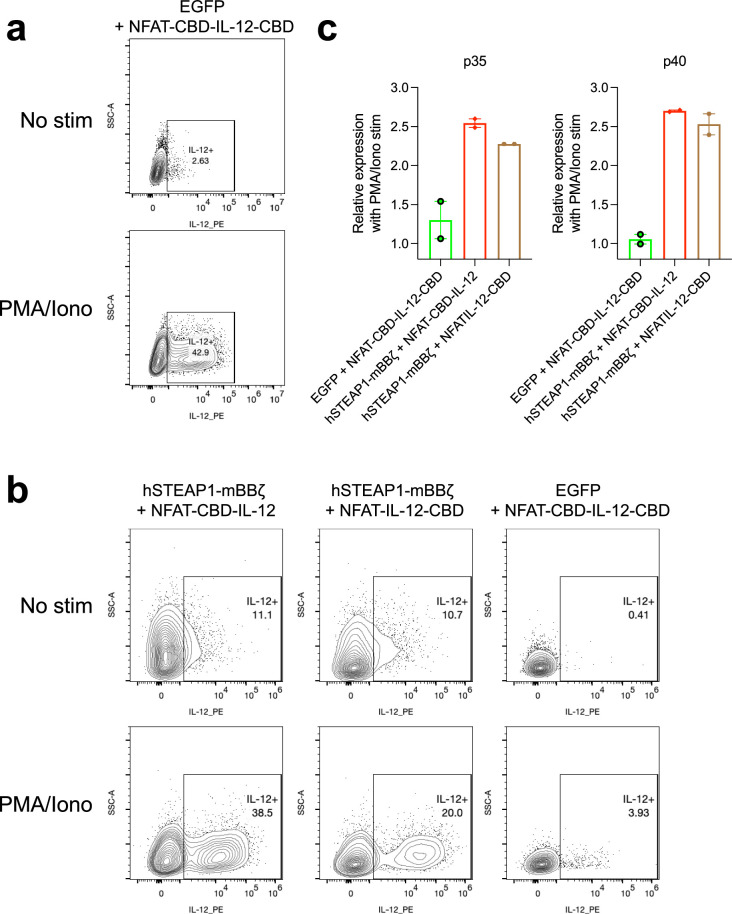
Translation and transcription of IL-12 variants in Jurkat and primary mouse T cells. (**a**) Jurkat cells transduced with EGFP + NFAT-CBD-IL-12-CBD gamma-retroviral vector were stimulated with PMA/Iono in the presence of Brefeldin A followed by intracellular staining of mouse IL-12. Representative contour plots of n = 2 technical replicates. (**b**) Primary mouse T cells transduced with the indicated gamma-retroviral vectors were stimulated with PMA/Iono in the presence of Brefeldin A followed by intracellular staining of mouse IL-12. Representative contour plots of n = 2 technical replicates. (**c**) Primary mouse T cells transduced with the indicated gamma-retroviral vectors were stimulated with PMA/Iono followed by RNA extraction, reverse transcription and quantitative PCR. Expression levels of mouse p35 and p40 in PMA/Iono stimulated cells relative to unstimulated cells were shown (n = 2 technical replicates). Source data

All the STEAP1 CAR-T variants lysed RM9-hSTEAP1-firefly luciferase (Fluc) mouse prostate cancer cells, but not RM9 wildtype (WT) (Fig. 1f and Supplementary Fig. 1a). Expression of the IL-12 variants significantly increased cytotoxicity against RM9-hSTEAP1 cells, suggesting that autocrine IL-12 contributes to the enhanced cytolytic capacity^10^. CAR-T cells armoured with one CBD fused to IL-12 (CBD-IL-12 or IL-12-CBD) secreted the payload upon stimulation (Fig. 1g). However, the amount of secreted CBD-IL-12-CBD was low. Although CBD-IL-12-CBD was produced in HEK293F and Jurkat cells (Fig. 1b and Extended Data Fig. 2a), its protein and mRNA expressions in primary mouse T cells were inefficient compared with other IL-12 variants (Extended Data Fig. 2b,c). Nonetheless, all the armoured CAR-T cells showed enhanced IFNγ production (Fig. 1h). These results demonstrate that the IL-12 variant-armoured hSTEAP1 CAR-T cells can lyse hSTEAP1-positive cancer cells more potently and secrete the payload in an antigen-dependent manner.

### CBD-IL-12-armoured hSTEAP1 CAR-T cells demonstrate anti-tumour efficacy without pre-conditioning

**Fig. 2:**
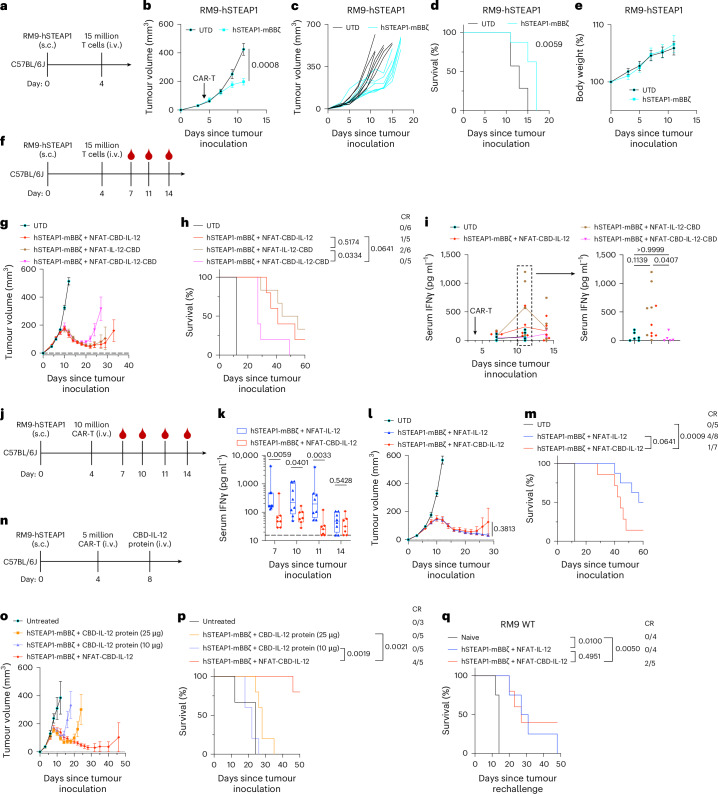
Characterizations of CAR-T cells armoured with a collagen-binding IL-12 in vivo. **a**–**e**, Male C57BL6/J mice received subcutaneous injection of RM9-hSTEAP1 (5 × 10^5^) on day 0. **a**, Experimental timeline; 15 million T cells were intravenously injected on day 4. **b**, Average tumour volumes (mean ± s.e.m.). **c**, Individual tumour growth curves. **d**, Survival rates. **e**, Body weight changes normalized to the body weights on day 0 (mean ± s.e.m.). **f**–**i**, **f**, Experimental timeline; 15 million T cells were intravenously injected on day 4 followed by blood sampling on days 7, 11 and 14. **g**, Average tumour volumes (mean ± s.e.m.). **h**, Survival rates. CR, complete response. **i**, Serum IFNγ concentrations were quantified by ELISA (mean). **j**–**m**, **j**, Experimental timeline; 10 million CAR-T cells were intravenously injected on day 4 followed by blood sampling on days 7, 10, 11 and 14. **k**, Serum IFNγ concentrations were quantified by ELISA. The boxes extend from the 25th to the 75th percentiles, the centre lines show median values and the whiskers extend to the minima and maxima. The dotted line shows the detection limit (15.6 pg ml^−1^). **l**, Average tumour volumes (mean ± s.e.m.). **m**, Survival rates. **n**–**p**, **n**, Experimental timeline; 5 million CAR-T cells were intravenously injected on day 4 followed by a single intravenous injection of CBD-IL-12 protein on day 8. **o**, Average tumour volumes (mean ± s.e.m.). **p**, Survival rates. **q**, Survival curves for complete responders (in **j**–**p**) subcutaneously rechallenged with RM9 WT cells (5 × 10^5^) on day 60. (**a**–**e**) UTD, *n* = 7 mice; hSTEAP1-mBBζ, *n* = 8. (**f**–**i**) hSTEAP1-mBBζ + NFAT-CBD-IL-12 and hSTEAP1-mBBζ + NFAT-CBD-IL-12-CBD, *n* = 5; UTD and hSTEAP1-mBBζ + NFAT-IL-12-CBD, *n* = 6. (**j**–**m**) UTD, *n* = 5; hSTEAP1-mBBζ + NFAT-IL-12, *n* = 8; hSTEAP1-mBBζ + NFAT-CBD-IL-12, *n* = 7. (**n**–**p**) *n* = 3 for untreated; *n* = 5 for all other groups. (**a**–**e**) 75.3% CAR^+^ in hSTEAP1-mBBζ. (**f**–**i**) 54.1% CAR^+^ in hSTEAP1-mBBζ + NFAT-CBD-IL-12, 62.8% CAR^+^ in hSTEAP1-mBBζ + NFAT-IL-12-CBD and 52.3% CAR^+^ in hSTEAP1-mBBζ + NFAT-CBD-IL-12-CBD. Statistical analyses were performed using two-tailed Welch’s *t*-test (**b**,**l**), log-rank (Mantel–Cox) test (**d**,**h**,**m**,**p**,**q**), Kruskal–Wallis test followed by Dunn’s multiple comparisons (**i**) or two-tailed Mann–Whitney test (**k**). *P* values are shown in figures. Source data

**Extended Data Fig. 3:**
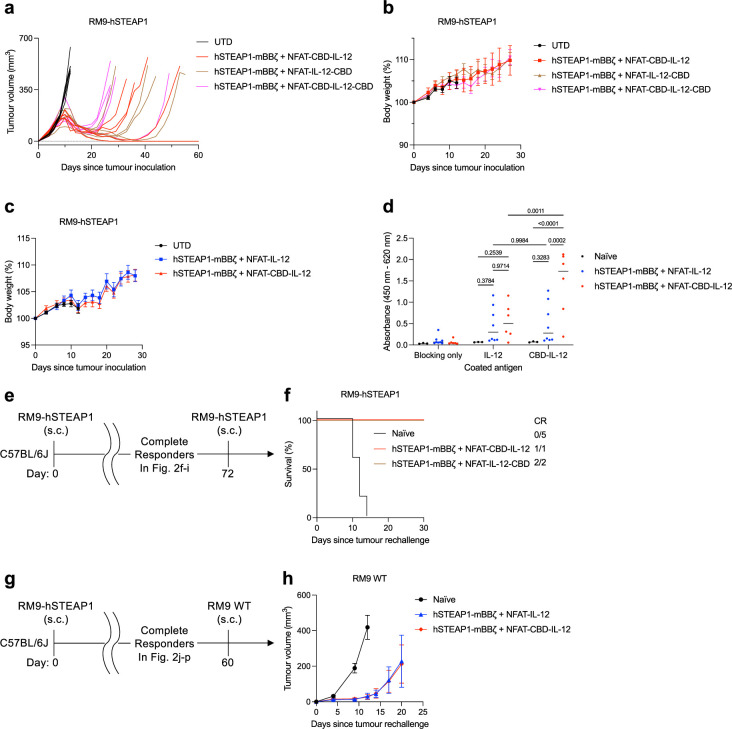
Additional in vivo data, related to Fig. 2. **a**) Individual tumour growth curves in Fig. 2g. (**b, c**) Body weight changes normalized to the body weights on day 0, related to (**b**) Fig. 2f–i and (**c**) Fig. 2j–m (mean ± s.e.m.). (**d**) Plasma samples were collected from survivors in Fig. 2j–m on day 35. Plasma IgG reactive to IL-12 and CBD-IL-12 was detected by ELISA (mean). (**e, f**) (**e**) Experimental timeline and (**f**) survival curves for RM9-hSTEAP1 rechallenge using complete responders in Fig. 2f–i. (**g, h**) (**g**) Experimental timeline and (**h**) average tumour volumes for RM9 WT rechallenge using complete responders in Fig. 2j–p. (**d**) Statistical analysis was performed using (**d**) two-way ANOVA followed by Šídák’s multiple comparisons. *P* values are shown in figures. Source data

We tested the therapeutic efficacy of the conventional hSTEAP1 CAR-T cells against subcutaneous RM9-hSTEAP1-Fluc tumour (Fig. 2a–e). As we previously reported, hSTEAP1 CAR-T cells showed significant anti-tumour effects (Fig. 2b–e). However, complete response (CR) was not observed. Next, we treated the same tumour model to screen the collagen-binding IL-12-armoured CAR constructs (Fig. 2f–i). All the armoured CAR-T treatments clearly delayed tumour growth (Fig. 2g and Extended Data Fig. 3a) and prolonged survival compared with untransduced (UTD) T cells (Fig. 2h). One of five mice treated with CBD-IL-12-armoured CAR-T (CBD-IL-12 CAR-T) cells showed CR, as did two of six mice treated with IL-12-CBD CAR-T cells, whereas tumours were not eradicated in any mice treated with CBD-IL-12-CBD CAR-T cells (Fig. 2h). Body weight loss was not observed (Extended Data Fig. 3b). There was a transient and non-statistically significant trend towards increased serum IFNγ levels in mice treated with CBD-IL-12 or IL-12-CBD CAR-T cells on day 11 (Fig. 2i). CBD-IL-12-CBD CAR-T treatment did not increase serum IFNγ levels, consistent with poor expression of CBD-IL-12-CBD (Fig. 1g and Extended Data Fig. 2). These results indicate that fusing one CBD to IL-12 is more therapeutically effective than fusing two CBDs.

### CAR-T-cell-mediated delivery of CBD-IL-12 balances therapeutic efficacy and safety

We selected N-terminal fusion CBD-IL-12-armoured CAR-T cells for detailed comparisons with IL-12-armoured CAR-T cells given the similar characteristics of N- and C-terminal fusions in vitro and in vivo. CBD fusion to IL-12 significantly decreased serum IFNγ levels in the treated mice (Fig. 2j,k). Body weight loss was not observed (Extended Data Fig. 3c). Albeit not statistically significant, IL-12 CAR-T cells showed stronger anti-tumour efficacy compared with CBD-IL-12 CAR-T cells (Fig. 2l,m). Immunogenicity of the CBD (human origin) might have disturbed long-term therapeutic efficacy of CBD-IL-12 CAR-T in the immunocompetent mouse model because we detected anti-CBD-IL-12 IgG in plasma (Extended Data Fig. 3d).

Next, we compared therapeutic efficacy of CBD-IL-12 hSTEAP1 CAR-T cells with the combination therapy of hSTEAP1 CAR-T cells and CBD-IL-12 protein (Fig. 2n)^22^. The monotherapy of CBD-IL-12 CAR-T cells outperformed the combination therapy and showed 80% of CR rate (Fig. 2o,p). This suggests that CAR-T-cell-mediated, local and continuous delivery of CBD-IL-12 to the TME is more efficacious than a single, large dose of the protein.

To investigate whether CBD-IL-12 CAR-T cells developed anti-tumour immune memory to hSTEAP1 and other antigens, we rechallenged the complete responders with RM9-hSTEAP1 or parental RM9 WT cells (hSTEAP1^−^). All the long-term survivors rejected the second challenge with RM9-hSTEAP1, demonstrating anti-tumour immune memory against hSTEAP1 (Extended Data Fig. 3e,f). RM9 WT tumours were also suppressed when implanted to the complete responders, and the survivors previously treated with CBD-IL-12 CAR-T cells showed 40% of rejection rate (Fig. 2q and Extended Data Fig. 3g,h). The results indicate that CBD-IL-12 CAR-T cells induce antigen spreading to counteract antigen heterogeneity.

### Secreted CBD-IL-12 demonstrates enhanced localization to the tumour

**Fig. 3:**
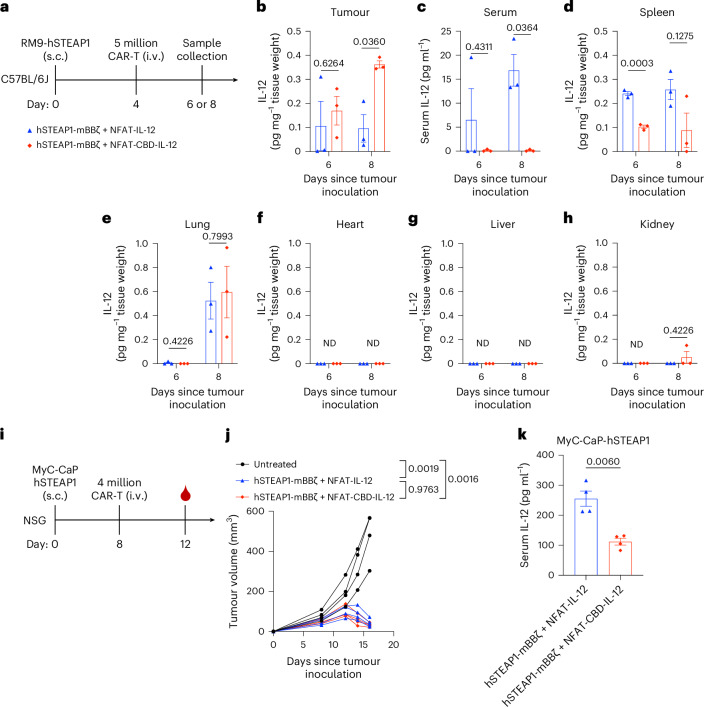
CBD fusion enhances intratumoural retention of IL-12 and reduces its systemic leakage. **a**–**h**, **a**, Experimental timeline. Male C57BL6/J mice received subcutaneous injection of RM9-hSTEAP1 (5 × 10^5^) on day 0; 5 million CAR^+^ T cells were intravenously administered on day 4. **b**, Tumours, **c**, sera and **d**–**h**, organs were collected at indicated timepoints in **a**. IL-12 was quantified by ELISA (*n* = 3 mice, mean ± s.e.m.). ND, not detected. **i**–**k**, **i**, Experimental timeline. Male NSG mice received subcutaneous injection of MyC-CaP-hSTEAP1 (5 × 10^5^) on day 0; 4 million CAR^+^ T cells (derived from male C57BL6/J mice) were intravenously administered on day 8 followed by blood sampling on day 12 (*n* = 4 mice for all groups). **j**, Individual tumour growth curves. **k**, Serum IL-12 concentrations were quantified by ELISA (biological replicates, mean ± s.e.m.). Statistical analyses were performed using two-tailed Welch’s *t*-test (**b**–**e**,**h**,**k**) or one-way ANOVA with Tukey’s test (on day 16) (**j**). *P* values are shown in figures. Source data

**Extended Data Fig. 4:**
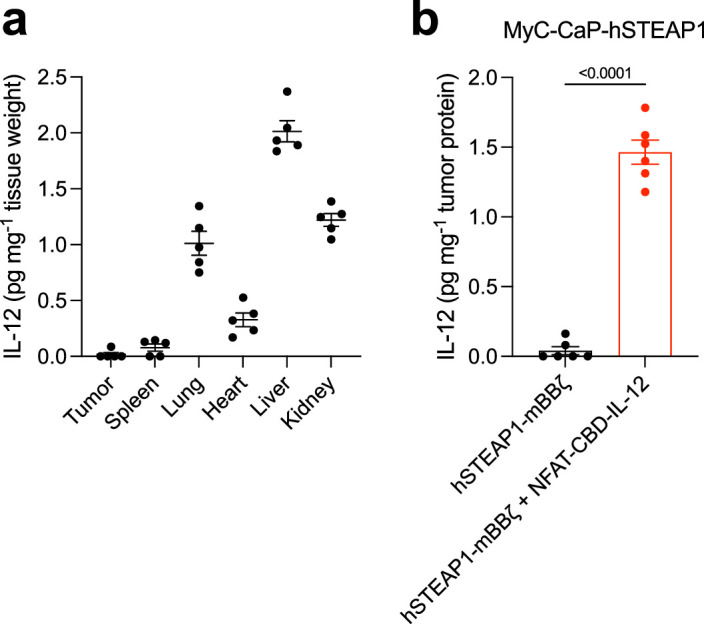
Detection of IL-12 from tumour and organs, related to Fig. 3. (**a**) Background concentration of IL-12 in major organs from RM9-hSTEAP1 tumour-bearing mice treated with 10 million UTD T cells (organs were collected when tumour volume reached the endpoint), normalized by total tissue weight (each dot represents a mouse, mean ± s.e.m.). (**b**) FVB/N male mice bearing subcutaneous MyC-CaP-hSTEAP1 tumours (200–270 mm^3^) were intravenously injected with 5 million CAR-T cells. Tumours were collected after 4 days and intratumoural IL-12 concentration was quantified by ELISA and normalized by total protein content (n = 2 tumours × 3 fragments = 6 replicates for each group, mean ± s.e.m.). Statistical analysis was performed using (**b**) two-tailed Welch’s t-test. *P* values are shown in figures. Source data

We quantified the IL-12 and CBD-IL-12 secreted from CAR-T cells in vivo (Fig. 3a). Without the administration of IL-12-armoured CAR-T cells, IL-12 was barely detectable in the tumour (Extended Data Fig. 4a,b). On day 8, a significantly higher level of intratumoural IL-12 was detected in mice treated with CBD-IL-12 CAR-T cells (Fig. 3b). In contrast, serum concentrations of IL-12 were significantly lower in CBD-IL-12 CAR-T-treated mice on day 8 (Fig. 3c). Significantly less IL-12 was detected from spleens of CBD-IL-12 CAR-T-treated mice on day 6 (Fig. 3d). The levels of IL-12 detected from the lung were comparable between the two groups (Fig. 3e). IL-12 was barely detectable in the heart, liver and kidney (Fig. 3f–h). Given that the detected concentrations of IL-12 in serum were low in the immune competent model, we next quantified CAR-T-derived IL-12 using NSG mice, in which endogenous immune cells that consume IL-12 are scarce. MyC-CaP-hSTEAP1-bearing male NSG mice received C57BL6/J-derived CAR-T cells intravenously (Fig. 3i). Both CBD-IL-12 CAR-T cells and IL-12 CAR-T cells showed anti-tumour efficacy (Fig. 3j). Importantly, serum CBD-IL-12 level was significantly lower than serum IL-12 level (Fig. 3k). These results indicate that CBD-IL-12 secreted from CAR-T cells are significantly more localized to the tumour and less distributed to other parts of the body compared with IL-12.

### CBD-IL-12 CAR-T therapy decreases systemic toxicity

**Fig. 4:**
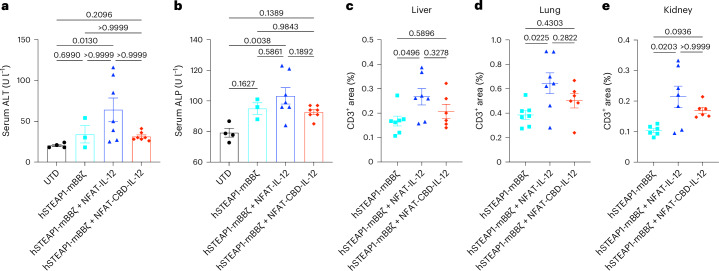
CBD decreases IL-12-related toxicity of armoured CAR-T cells. Male C57BL6/J mice received subcutaneous injection of RM9-hSTEAP1 (5 × 10^5^) on day 0. CAR-T cells were intravenously administered on day 4. **a**, Serum ALT and **b**, ALP concentrations on day 12 (each dot represents a mouse, mean ± s.e.m.). **c**–**e**, Immunohistochemistry quantification of CD3^+^ T cell infiltration in liver (**c**), lung (**d**) and kidney (**e**) on day 14 (each dot represents a mouse, mean ± s.e.m.). Statistical analyses were performed using Kruskal–Wallis test followed by Dunn’s multiple comparisons (non-parametric data) (**a**,**e**) or one-way ANOVA with Tukey’s test (**b**–**d**). *P* values are shown in figures. Source data

**Extended Data Fig. 5:**
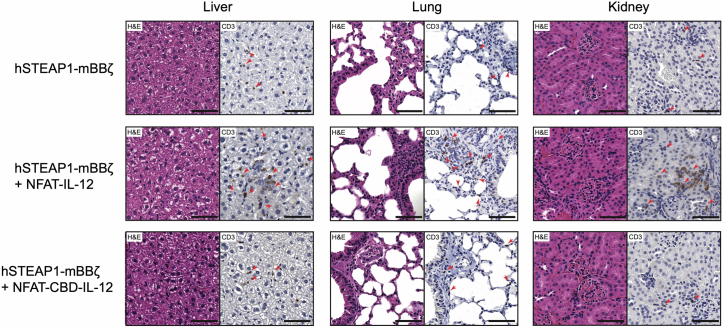
Histological analysis of liver, lung and kidney, related to Fig. 4. Representative H&E and IHC (CD3) staining images from an experiment. Red arrows refer to CD3^+^ cells. Scale bars, 50 µm.

irAEs are one of the major obstacles for clinical translation of IL-12 therapies. Hepatic toxicity is a common side effect in patients who receive systemic administration of recombinant human IL-12 (ref. ^25^). Serum concentrations of liver damage-associated ALT and alkaline phosphatase (ALP) were significantly increased by IL-12 CAR-T cells, but not by CBD-IL-12 CAR-T cells (Fig. 4a,b and Supplementary Fig. 2a). IL-12 CAR-T cells significantly facilitated infiltration of CD3^+^ T cells into non-target organs (liver, lung and kidney) compared with CAR-T cells, whereas CBD-IL-12 CAR-T cells did not (Fig. 4c–e, Supplementary Fig. 2b and Extended Data Fig. 5). No structural abnormalities were found in organs of any treatment group in haematoxylin and eosin (H&E) staining (Extended Data Fig. 5). These results demonstrate that the CBD fusion to IL-12 reduces the toxicity of CAR-T cells armoured with IL-12.

### CBD-IL-12 CAR-T cells inflame tumours and induce anti-tumour immune infiltrates

**Fig. 5:**
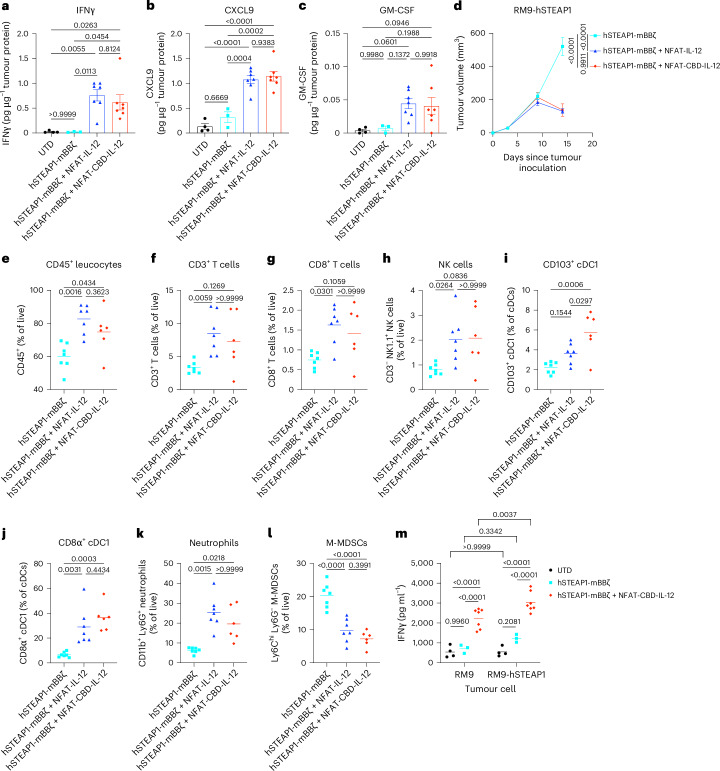
CBD-IL-12-armoured CAR-T cells exert an anti-tumour effect by fuelling innate and adaptive immunity. Male C57BL6/J mice received subcutaneous injection of RM9-hSTEAP1 (5 × 10^5^) on day 0. CAR-T cells were intravenously administered on day 4. **a**–**c**, Intratumoural concentrations of IFNγ (**a**), CXCL9 (**b**) and GM-CSF (**c**) on day 12 (each dot represents a mouse, mean ± s.e.m.). **d**–**l**, Tumours were collected on day 14 followed by flow cytometric analysis. **d**, Tumour volumes (mean ± s.e.m.). hSTEAP1-mBBζ, hSTEAP1-mBBζ + NFAT-IL-12, *n* = 7; hSTEAP1-mBBζ + NFAT-CBD-IL-12, *n* = 6. **e**, Percentage of CD45^+^ cells, **f**, CD3^+^ T cells, **g**, CD8^+^ T cells and **h**, CD3^−^ NK1.1^+^ NK cells within live cells (mean). Percentage of **i**, CD103^+^ and **j**, CD8α^+^ cDC1 among cDCs. Percentage of **k**, CD11b^+^ Ly6G^+^ neutrophils and **l**, Ly6C^hi^ Ly6G^−^ M-MDSCs within live cells (each dot represents a mouse, and the centre lines show mean values). **m**, Splenic T cells were isolated and co-cultured with hSTEAP1-positive and negative RM9 prostate cancer cells. IFNγ was quantified (each dot represents a mouse, and centre lines show mean values). Statistical analyses were performed using one-way ANOVA with Tukey’s test (**a**–**e**,**i**,**j**,**l**), Kruskal–Wallis test followed by Dunn’s multiple comparisons (non-parametric data) (**f**–**h**,**k**) or two-way ANOVA followed by Šídák’s multiple comparisons (**m**). *P* values are shown in figures. Source data

Tumour cell heterogeneity is the major cause of recurrence and inefficacy of CAR-T cells. We observed CRs in some of the animals (Fig. 2h,m,p) as well as rejection of RM9 WT rechallenge in a fraction of the complete responders (Fig. 2q), suggesting that CBD-IL-12 CAR-T cells induce antigen spreading. We analysed the TME to investigate the underlying mechanisms. IL-12-induced IFNγ promotes cross-presentation by upregulating expressions of key proteins, such as MHC-I^11^. CXCL9 recruits T cells and NK cells into the TME^26^. The granulocyte macrophage colony-stimulating factor (GM-CSF) is also induced by IL-12, facilitating the maturation of cross-presenting DCs^12^ that have a major role in T cell-mediated anti-tumour immunity^27,28^. We found that IL-12 CAR-T cells and CBD-IL-12 CAR-T cells upregulate intratumoural IFNγ, CXCL9 and GM-CSF compared with UTD and unarmoured CAR-T cells (Fig. 5a–c and Supplementary Fig. 2a).

**Extended Data Fig. 6:**
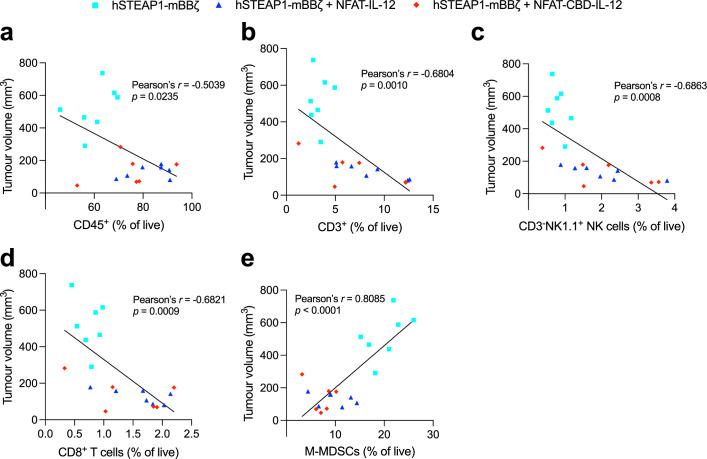
Correlation analysis between tumour volume and frequency of immune cells. Simple linear regression and correlation analyses between tumour volume and (**a**) %CD45^+^ leukocytes, (**b**) %CD3^+^ T cells, (**c**) %CD3^−^ NK1.1^+^ NK cells, (**d**) %CD3^+^ CD8^ +^ T cells or (**e**) %Ly6C^hi^ Ly6G^−^ M-MDSCs within live cells were performed using flow cytometric data presented in Fig. 5. Each dot represents a mouse. Pearson correlation coefficient and two-tailed *P* values are shown. Source data

We also characterized the immune cell infiltrates in the RM9-hSTEAP1 tumour by flow cytometry (Fig. 5d–l and Supplementary Fig. 2b). In accordance with the increase in intratumoural CXCL9, CBD-IL-12 CAR-T cells increased the frequency of CD45^+^ leucocytes, CD3^+^ T cells, CD8^+^ T cells and CD3^−^NK1.1^+^ NK cells compared with CAR-T cells (Fig. 5e–h), and the increased frequencies correlated with a lower tumour burden (Extended Data Fig. 6a–d). CBD-IL-12 CAR-T cells increased cross-presenting conventional type 1 DCs (cDC1s), including CD103^+^ cDC1s and CD8α^+^ cDC1s within the CD11c^+^MHC-II^+^ cDC population (Fig. 5i,j), in accordance with the increase in GM-CSF. CBD-IL-12 CAR-T cells significantly increased the frequency of neutrophils, which play a crucial role in eliminating antigen-negative cancer cells following adoptive transfer of antigen-specific T cells^29^ (Fig. 5k). CBD-IL-12 CAR-T cells significantly reduced CD11b^+^Ly6C^hi^Ly6G^−^ monocytic MDSCs (M-MDSCs), an especially suppressive subset of MDSCs^30^ (Fig. 5l), and M-MDSC frequencies positively correlated with tumour burdens (Extended Data Fig. 6e). Splenic T cells derived from mice treated with CBD-IL-12 CAR-T cells produced a significantly higher amount of IFNγ when co-cultured with RM9 WT (Fig. 5m), suggesting T cell responses to antigens other than hSTEAP1. Collectively, CBD-IL-12 CAR-T cells change the composition of immune infiltrates in the tumour towards an anti-tumour state, inducing antigen spreading to fight against heterogeneity of the prostate cancer.

### CBD-IL-12 CAR-T cells remodel the intratumoural transcriptional landscape to enhance anti-tumour immunity

**Fig. 6:**
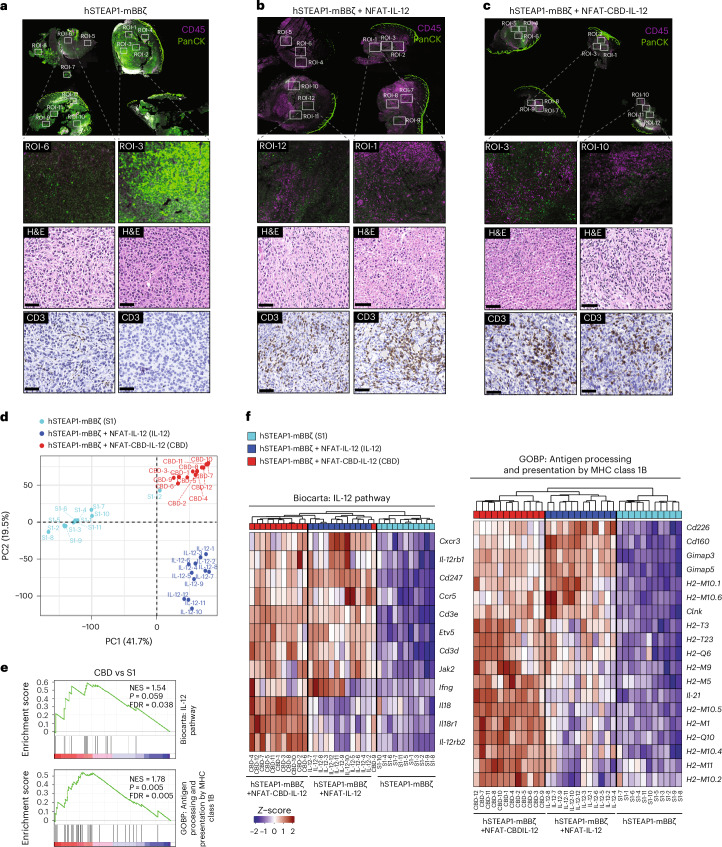
Spatial transcriptome analysis shows CBD-IL-12 CAR-T-mediated changes in the tumour microenvironment. **a**–**c**, Regions of interest from hSTEAP1-mBBζ (**a**) (*n* = 3 mice), hSTEAP1-mBBζ + NFAT-IL-12 (**b**) (*n* = 4) and hSTEAP1-mBBζ + NFAT-CBD-IL-12 (**c**) (*n* = 4) treated mice. Immunofluorescence image depict pan-cytokeratin (panCK, green) and CD45 (magenta) stain. Representative images of sections stained with H&E and CD3 are also shown. Scale bars, 50 µm. **d**, PCA plot showing the distribution of selected ROIs colour coded on the basis of treatment groups. **e**, Gene set enrichment analysis showing enriched IL-12 pathway and antigen processing and presentation via MHC-class 1B. **f**, Heat maps showing changes in genes involved in pathways as in **e**. (**e**) Enrichment *P* values were computed using phenotype-based permutation tests (*n* = 1,000 permutations), and false discovery rate (FDR) *q*-values were calculated using Benjamini–Hochberg multicomparison analysis.

**Extended Data Fig. 7:**
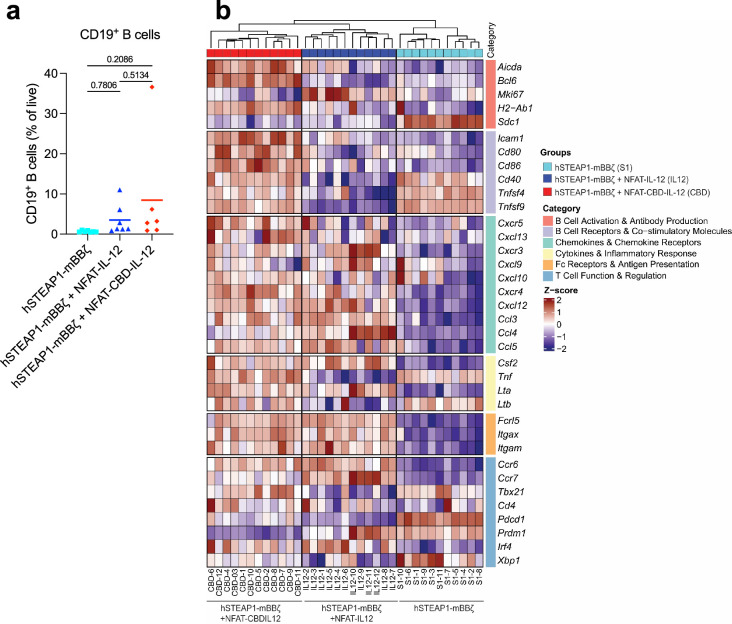
Flow cytometric and transcriptomic analyses of CBD-IL-12 CAR-T treated tumours, related to Fig. 6. (**a**) Tumours were collected according to Supplementary Fig. 2b for flow cytometric analysis. Percentage of CD19^+^ B cells within live cells (mean). (**b**) A heatmap showing transcriptional changes in genes involved in tertiary lymphoid structure. Statistical analysis was performed using (**a**) one-way ANOVA with Tukey’s test. Source data

Limited trafficking and infiltration of CAR-T cells in solid tumours and immunosuppressive TME are major challenges to the efficacy of current therapies. We performed spatial transcriptomics to assess whether the entire TME can be remodelled by CBD-IL-12 CAR-T cells (Supplementary Fig. 2b). We observed infiltration of CD45^+^ leucocytes throughout the tissues and reduction of tumour regions (pan-cytokeratin^+^) in IL-12 CAR-T or CBD-IL-12 CAR-T-treated samples (Fig. 6a–c). Immunohistochemistry (IHC) staining confirmed enhanced infiltration of CD3^+^ T cells in the armoured CAR-T-treated tumours. Principal component analysis (PCA) showed that tumour tissues treated with the three different therapies displayed markedly different characteristics (Fig. 6d). Gene set enrichment analysis (GSEA) revealed that CBD-IL-12 CAR-T cells activated the IL-12 pathway and activated antigen processing and presentation by MHC-I (Fig. 6e,f). Enrichment of genes in the IL-12 pathway was not statistically significant (*P* = 0.059), presumably because only 17 genes in the dataset were annotated to this pathway. Indeed, Fisher’s exact test showed statistical significance of IL-12 pathway enrichment in differentially expressed genes (*P* = 0.01241). Because we observed a tendency for an increase in B cells in tumours treated with IL-12 CAR-T cells and CBD-IL-12 CAR-T cells (Extended Data Fig. 7a), we analysed gene expression changes related to tertiary lymphoid structure (TLS) (Extended Data Fig. 7b). We observed increased expression of a variety of TLS-related chemokines and chemokine receptors including *Cxcr4, Cxcr5, Cxcl12* and *Cxcl13* in both CBD-IL-12 CAR-T and IL-12 CAR-T-treated tumours. Interestingly, CBD-IL-12 CAR-T cells, but not IL-12 CAR-T cells, clearly upregulated *Bcl6* (master regulator for T follicular helper cells^31–33^) and *Aicda* (essential for class switch recombination and somatic hypermutation^34,35^). Consistent with the upregulation of *Bcl6*, *Prdm1* (master regulator of plasma cells^36^ and known to reciprocally regulate *Bcl6* (ref. ^31^)) was downregulated by CBD-IL-12 CAR-T cells. These data suggest that CBD-IL-12 CAR-T cells enhanced the maturation and function of intratumoural immune aggregates resembling TLS. We also observed specific enrichment of co-stimulatory molecules such as *Cd80*, *Cd86*, *Cd40* and *Tnfsf4* in the CBD-IL-12 CAR-T-treated tumours. These co-stimulatory signals may facilitate local T cell priming and promote immune synapse formation, thereby enhancing anti-tumour immunity. Collectively, the data suggest that CBD-IL-12 CAR-T cells reprogramme the TME towards a more immunologically active, TLS-supportive niche.

### CBD-IL-12 CAR-T cells combined with immune checkpoint inhibitors (CPIs) eradicate large established RM9-hSTEAP1 tumours

**Fig. 7:**
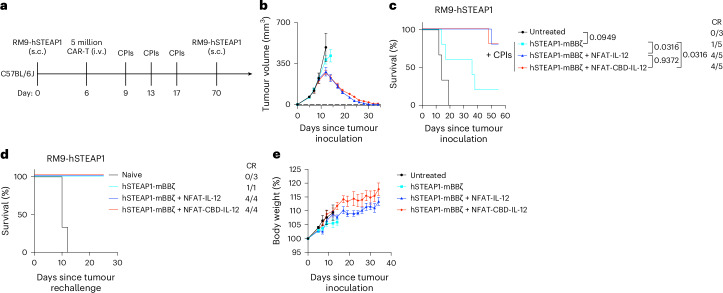
CBD-IL-12-armoured CAR-T cells eradicate established RM9-hSTEAP1 tumour in combination with anti-PD-1 and anti-CTLA-4 checkpoint inhibitors. **a**, Experimental timeline; 5 million CAR^+^ T cells were intravenously administered on day 6. Anti-PD-1 and anti-CTLA-4 antibodies were administered intraperitoneally 3 times starting at 3 days after CAR-T administration with 4 days interval. Untreated, *n* = 3 mice; all the other groups, *n* = 5 mice. **b**, Tumour volumes (mean ± s.e.m.). **c**, Survival rates. **d**, Complete responders were subcutaneously rechallenged with RM9-hSTEAP1 cells (5 × 10^5^) on day 70. **e**, Body weight changes normalized to body weights on day 0 (mean ± s.e.m.). Statistical analysis was performed using log-rank (Mantel–Cox) test (**c**). *P* values are shown in figures. Source data

**Extended Data Fig. 8:**
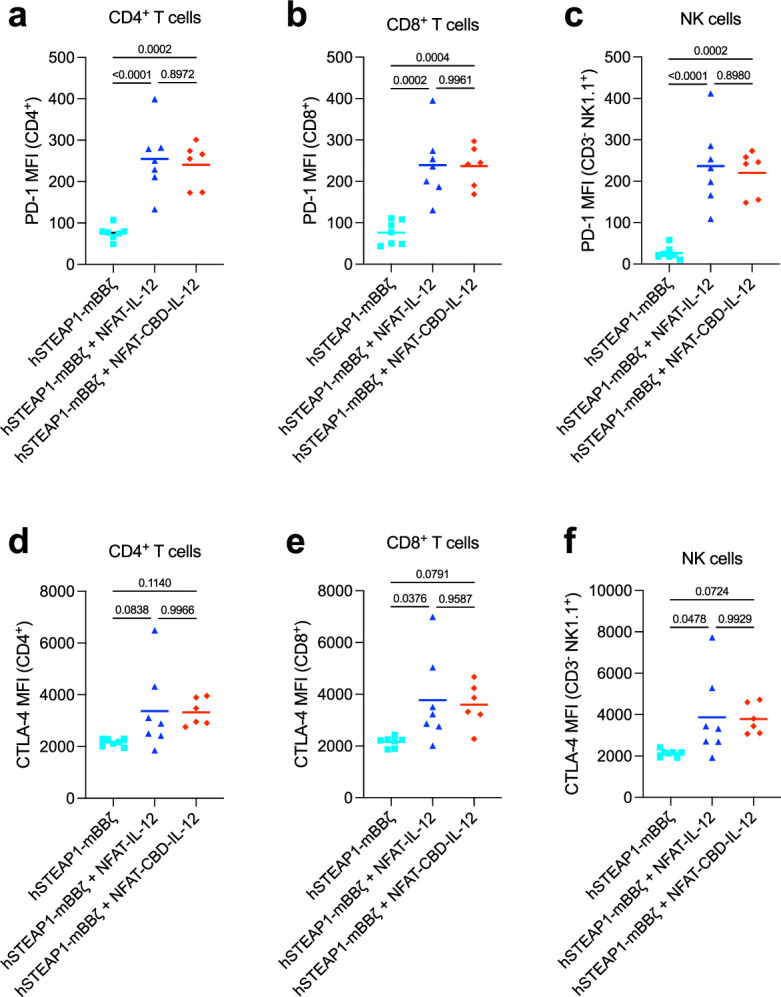
Expression levels of PD-1 and CTLA-4 in tumour-infiltrating T and NK cell compartments following armoured CAR-T therapy. Male C57BL6/J mice received subcutaneous injection of RM9-hSTEAP1 (5×10^5^) on day 0. 5 million CAR^+^ T cells were intravenously administered on day 4. Tumours were collected on day 14 followed by flow cytometric analysis. (**a–c**) Median fluorescence intensity (MFI) indicating PD-1 expression levels in (**a**) CD3^+^ CD4^+^ , (**b**) CD3^+^ CD8^+^ and (**c**) CD3^−^ NK1.1^+^ cells are shown (mean). (**d–f**) MFI indicating CTLA-4 expression levels in (**d**) CD3^+^ CD4^+^ , (**e**) CD3^+^ CD8^+^ and (**f**) CD3^−^ NK1.1^+^ cells are shown (mean). Each dot represents a mouse. Statistical analyses were performed using one-way ANOVA with Tukey’s test. *P* values are shown in figures. Source data

**Extended Data Fig. 9:**
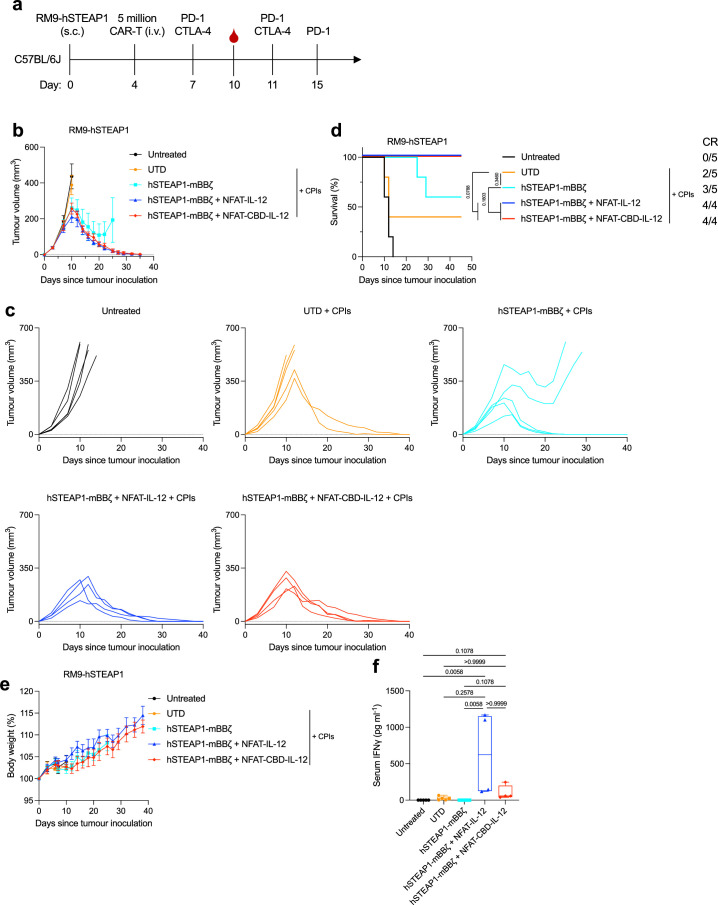
CBD-IL-12 armoured CAR-T cells eradicates RM9-hSTEAP1 tumour in combination with CPI. (**a**) Experimental timeline. Male C57BL6/J mice received subcutaneous injection of RM9-hSTEAP1 (5×10^5^) on day 0. Mice were left untreated or treated with combination immunotherapies of CAR-T cells and CPIs. 5 million CAR^+^T cells were intravenously administered on day 4. 100 µg of Anti-PD-1 antibody was administered intraperitoneally on days 7, 11 and 15. 100 µg of anti-CTLA-4 antibody was administered on days 7 and 11. (**b**) Tumour volumes were measured (Untreated, UTD and hSTEAP1-mBBζ, n = 5; hSTEAP1-mBBζ + NFAT-IL-12 and hSTEAP1-mBBζ + NFAT-CBD-IL-12, n = 4. mean ± s.e.m.). (**c**) Individual tumour growth curves. (**d**) Survival rates. CR, complete response. (**e**) Body weight changes normalized to the body weights on day 0 (mean ± s.e.m.). (**f**) Serum IFNγ concentrations on day 10. The boxes extend from 25th to 75th percentiles, the center lines show median values and the whiskers extend to the minima and maxima. Statistical analyses were performed using (**d**) log-rank (Mantel-Cox) test or (**f**) Kruskal-Wallis test followed by Dunn’s multiple comparison (non-parametric data). *P* values are shown in figures. Source data

Since CBD-IL-12 CAR-T cells alone did not constantly induce tumour-free CRs (Fig. 2h,m,p) and tumour-infiltrating T/NK cells had elevated programmed cell death protein 1 (PD-1) and cytotoxic T lymphocyte associated protein 4 (CTLA-4) expression (Extended Data Fig. 8a–f), we examined the anti-tumour efficacy of CBD-IL-12 CAR-T cells, anti-PD-1 and anti-CTLA-4 immune checkpoint inhibitors (CPIs) combination therapy. When the therapeutic intervention was started on day 4 (tumour volumes were ~60 mm^3^), armoured CAR-T cells in combination with the CPIs achieved a 100% CR rate (Extended Data Fig. 9a–d). Even when we delayed the initial CAR-T treatment until day 6 (tumour volumes were ~120 mm^3^), CBD-IL-12 CAR-T cells + CPIs cured 80% of RM9-hSTEAP1-bearing mice and significantly extended their survival compared with CAR-T cells + CPIs (Fig. 7a–c). These data show strong anti-tumour efficacy of the combination immunotherapy against the large established tumours. All the tumour-free survivors rejected subcutaneously rechallenged RM9-hSTEAP1 cells (Fig. 7d). None of the treatments caused body weight loss in the animals (Fig. 7e and Extended Data Fig. 9e). A previous study using IFNγ receptor-knockout (KO) mice indicated that IL-12-induced IFNγ plays a central role in irAEs^37^. In this study, significantly increased serum IFNγ was observed in mice treated with IL-12 CAR-T + CPIs, but not in mice treated with CBD-IL-12 CAR-T + CPIs (Extended Data Fig. 9f), consistent with the toxicity evaluation of the CAR-T monotherapies (Figs. 2k and 4a–e).

### Fully human CBD-IL-12 CAR-T cells suppress human prostate cancer xenograft and minimize circulating IL-12

**Fig. 8:**
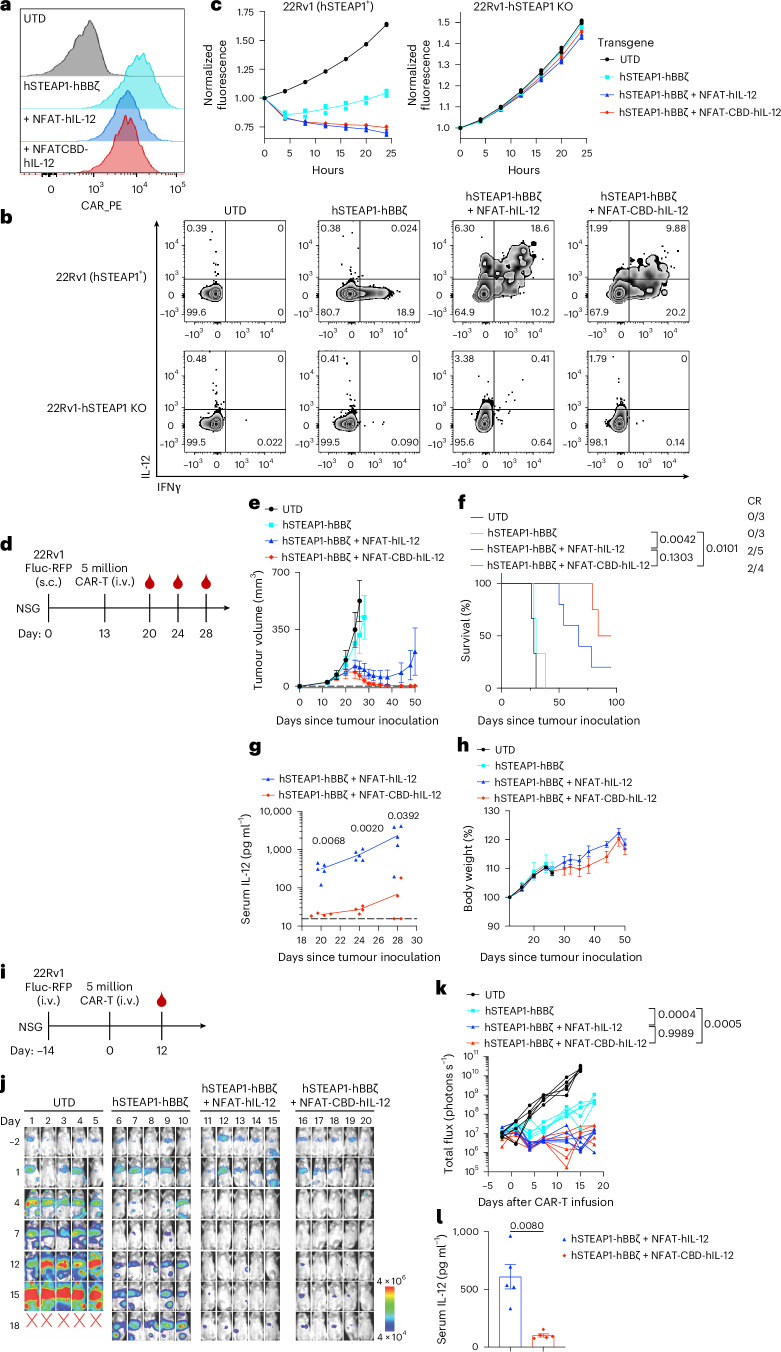
Primary human CBD-IL-12 CAR-T cells efficiently treat 22Rv1 human prostate cancer xenograft and reduce systemic IL-12. **a**, CAR expression on expanded primary human T cells was detected on the same day of the functional assays by flow cytometry (representative histograms from 2 biological replicates). **b**, Cytokine production upon overnight co-culture with target cells was detected with intracellular flow staining (representative zebra plots from 3 technical replicates). **c**, In vitro killing activity of human CD8 T cells transduced with indicated transgenes against hSTEAP1-positive or negative human prostate cancer target cells. Values from 3 technical replicate (dots) and mean values (lines) are shown. **d**–**h**, **d**, Experimental timeline. Male NSG mice received subcutaneous injection of 22Rv1-Fluc-RFP (2 × 10^6^) on day 0; 5 million CAR^+^ T cells were intravenously administered on day 13 followed by blood sampling on days 20, 24 and 28 (UTD, hSTEAP1-hBBζ, *n* = 3 mice; hSTEAP1-hBBζ + NFAT-hIL-12, *n* = 5; hSTEAP1-hBBζ + NFAT-CBD-hIL-12, *n* = 4). **e**, Average tumour volumes (mean ± s.e.m.). **f**, Survival rates. A complete responder previously treated with hSTEAP1-hBBζ + NFAT-hIL-12 was culled on day 78 due to GVHD-like symptoms. **g**, Serum IL-12 concentrations were quantified by ELISA (mean). The dotted line shows the detection limit (15.6 pg ml^−1^). **h**, Body weight changes normalized to body weights on day 0 (mean ± s.e.m.). **i**–**l**, **i**, Experimental timeline. Male NSG mice received intravenous injection of 22Rv1-Fluc-RFP (1 × 10^6^) on day −14; 5 million CAR^+^ T cells (at a CD4:CD8 ratio of 1:1) were intravenously administered on day 0 (*n* = 5 mice for all groups). **j**, Serial bioluminescence imaging of NSG mice engrafted with 22Rv1-Fluc-RFP metastases. Radiance scale is shown. **k**, Quantification of total flux images over time. **l**, Serum IL-12 concentration on day 12 (mean ± s.e.m.). Statistical analyses were performed using log-rank (Mantel–Cox) test (**f**), two-tailed Welch’s *t*-test (**g**,**l**) or one-way ANOVA with Tukey’s test (on day 18) (**k**). *P* values are shown in figures. Source data

**Extended Data Fig. 10:**
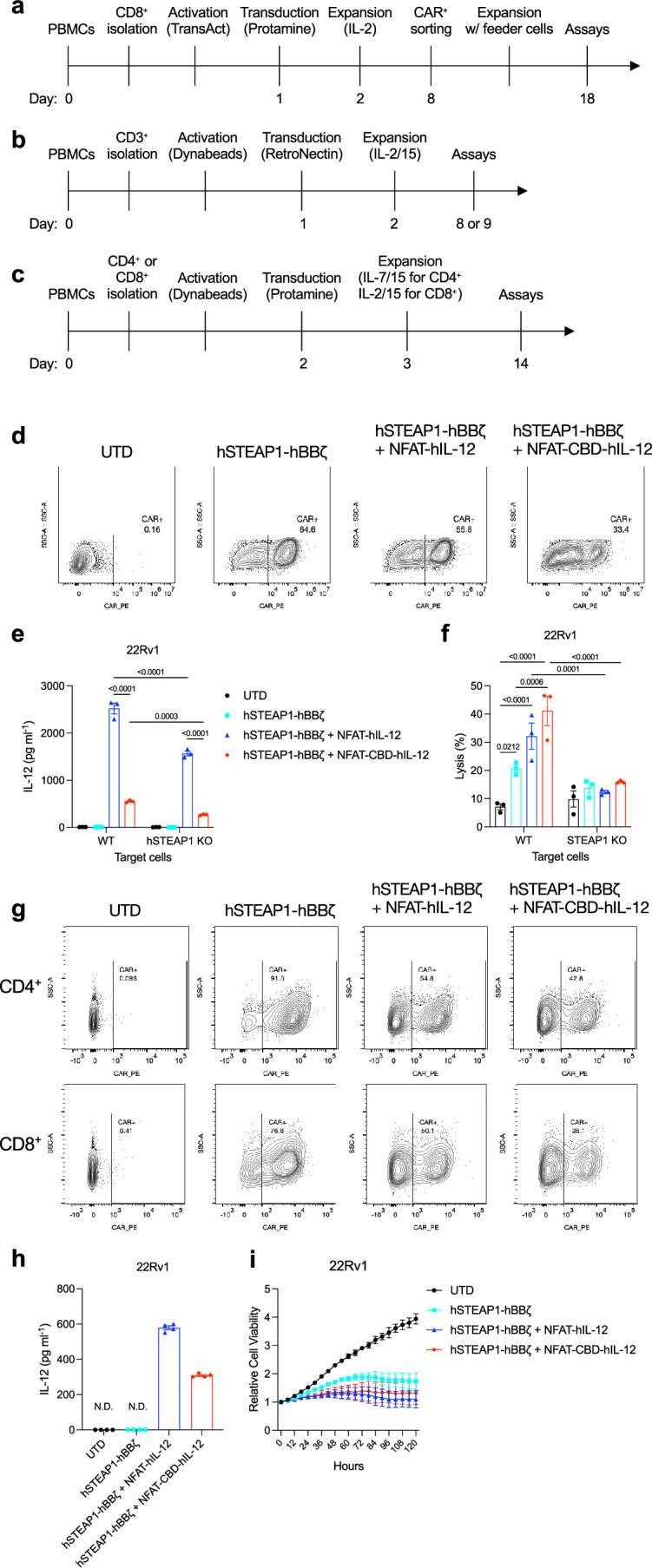
Manufacturing protocols and in vitro characterizations of primary human CAR-T cells, related to Fig. 8. (**a–c**) Experimental timelines for CAR-T cells used in (**a**) Fig. 8a–c, (**b**) Fig. 8d–h, Extended Data Fig. 10d-f and (**c**) Fig. 8i–l, Extended Data Fig. 10g-i. (**d**) Representative contour plots of primary human CAR-T cells stained with Biotin-goat anti-Human IgG and PE Streptavidin from two experiments. (**e**) Primary human CAR-T cells (50,000 CAR-T cells/well) were stimulated with 22Rv1 cells or 22Rv1 hSTEAP1 KO cells for 24 h. Secreted IL-12 variants were quantified by ELISA (technical replicates, mean ± s.e.m.). (**f**) 22Rv1 cells or 22Rv1 hSTEAP1 KO cells labelled with Calcein-AM were co-cultured with CAR-T cells at the Effector/Target ratio of 1:1 for 24 h. Calcein released from the target cells were quantified to calculate %target cell lysis (technical replicates, mean ± s.e.m.). (**g**) Representative contour plots of primary human CAR-T cells stained with Biotinylated Protein L and PE Streptavidin from an experiment. CD4 ^+^ CAR-T cells and CD8 ^+^ CAR-T cells were separately analysed. (**h, i**) Primary human CAR-T cells were co-cultured with 22Rv1 at Effector/Target ratio of 1:2.5. (**h**) IL-12 secretion from CAR-T cells (technical replicates, mean ± s.e.m.). (**i**) Relative cell viability of 22Rv1 target cells over time measured by fluorescence live cell imaging (n = 5 technical replicates, Mean ± s.d.). Statistical analyses were performed using (**e, f**) two-way ANOVA followed by Šídák’s multiple comparisons. *P* values are shown in figures. Source data

After confirming that unmodified and CBD-fused human IL-12 (CBD-hIL-12) proteins could be recombinantly expressed (Supplementary Fig. 3), we generated lentiviral vectors for human T cells. We successfully transduced Jurkat cells and primary human T cells with the hSTEAP1-hBBζ + NFAT-CBD-hIL-12 vector (Fig. 8a, Supplementary Fig. 4a,b and Extended Data Fig. 10a–d,g). Human CBD-hIL-12 CAR-T cells and hIL-12 CAR-T cells showed enhanced expression of hIL-12 upon co-culture with hSTEAP1^+^ 22Rv1 human prostate cancer cells (Fig. 8b and Extended Data Fig. 10e,h). These hIL-12-armoured CAR-T cells showed better in vitro killing against 22Rv1 compared with CAR-T cells without killing 22Rv1 hSTEAP1 KO cells (Fig. 8c and Extended Data Fig. 10f,i). Finally, we evaluated the human armoured CAR-T cells in subcutaneous (Fig. 8d–h) and disseminated metastatic 22Rv1 tumour models (Fig. 8i–l). STEAP1 CAR-T cells were unable to control the subcutaneous tumour growth (Fig. 8e,f). In contrast, both hIL-12 CAR-T cells and CBD-hIL-12 CAR-T cells induced tumour regression and prolonged survival. Strikingly, serum concentrations of CBD-hIL-12 were more than 10× lower than those of IL-12 at all timepoints tested (Fig. 8g). Body weight loss was not observed (Fig. 8h). Consistent with the subcutaneous model, serial bioluminescence imaging (BLI) demonstrated that hIL-12 CAR-T cells and CBD-hIL-12 CAR-T cells exhibited superior anti-tumour efficacy compared with CAR-T cells in the 22Rv1 disseminated tumour model (Fig. 8i–k). CBD fusion to hIL-12 significantly decreased serum IL-12 levels (Fig. 8l). Collectively, these results indicate that the CBD-IL-12-expression platform technology is applicable to human T cells.

## Discussion

The goal of cancer immunotherapy is to achieve safe and long-term effectiveness. The next milestone for CAR-T therapies is to overcome solid tumour unresponsiveness and/or tumour recurrence. We have previously reported that second-generation STEAP1 CAR-T cells induce tumour regression but face recurrence due to STEAP1 antigen loss. This could be overcome by combining with a potent cytokine, such as IL-12, which would efficiently activate the host immune system. We previously reported that conjugation of vWF-derived CBD to CPI antibodies (PD-L1, CTLA-4), cytokines (IL-2 and IL-12), chemokine CCL4 or serum albumin improves their localization to the tumour stroma, enhancing therapeutic efficacy and decreasing side effects^21,22,38,39^. Using our drug delivery approach that combines protein engineering and cell engineering, CBD-IL-12 expression from CAR-T cells showed stronger anti-tumour efficacy than just combining CAR-T with CBD-IL-12 protein, presumably due to local and sustained production of CBD-IL-12.

CBD-IL-12 expression from our novel CAR-T cell therapy improved the potency of CAR-T cells against mouse and human prostate cancer models by targeting the extracellular matrix. Compared with unmodified IL-12, the CBD fusion enhanced intratumoural retention of IL-12 from the CAR-T payload and reduced systemic IL-12 exposure, reducing irAEs. irAEs of IL-12-expressing adoptive T cell therapy is the major reason for clinical trial failures. The tumour-homing capacity of CBD-IL-12 (ref. ^22^) may contribute to toxicity reduction even when CBD-IL-12 is secreted from CAR-T cells in healthy tissues, due to the leakiness of the NFAT-responsive promoter and/or low-level STEAP1 expression in irrelevant tissues. Our data demonstrate that CBD fusion to IL-12 enhances the safety of IL-12 as a cargo of adoptive T cell therapies, and CBD-based drug delivery systems could be applied to other cell therapies.

Chemotherapy pre-conditioning is widely used in combination with CAR-T therapy to remove pre-existing immune cells and enable CAR-T cells to expand, infiltrate and remodel the TME^40^. While effective, chemotherapy often causes haematological and systemic toxicities and increased risks of frequent lethal infections^41^. In accordance with a previous study^17^, CBD-IL-12 CAR-T cells + CPI antibodies eradicated large established RM9-hSTEAP1 tumours without pre-conditioning. Given that IL-12 CAR-T cells combined with pre-conditioning failed because of toxicity in the clinic, it is worth investigating whether CBD-IL-12 CAR-T cells show objective response with tolerable toxicity in patients in the future.

One of the greatest challenges in the treatment of solid malignancies is antigen heterogeneity. IL-12 is a suitable cytokine for tackling this issue by enhancing the capacity of cellular immunity to fight diverse antigens. Our flow cytometric, transcriptomic and splenic T cell restimulation data suggest that CBD-IL-12 CAR-T cells can induce multifaceted activation of anti-tumour immunity and antigen spreading to fight antigen heterogeneity in solid tumours. The differences in gene expression patterns observed in the IL-12 CAR-T and CBD-IL-12 CAR-T groups in the transcriptomics analysis are intriguing. Investigating the mechanism by which these differences arise due to localization of IL-12, and how these differences can be leveraged to treat solid cancers including prostate cancer, would be our future work.

Other approaches to reduce toxicity of IL-12 as a payload include tethering IL-12 to the CAR-T cell surface by fusing a transmembrane domain (as we and others previously reported^42–44^) or an antibody fragment targeting cell surface receptors to IL-12 (ref. ^45^) and conjugating IL-12-loaded nanoparticles onto CAR-T cells through click chemistry^46^. Although these methods can be powerful in reducing toxicity, they might reduce the chance for IL-12 to act in *trans* by directly binding to an IL-12 receptor on endogenous immune cells. It will be important to see how these methodologies may differ from secreted IL-12 in terms of levels of therapeutic efficacy and mechanisms of action.

Our matrix-binding technology has several additional advantages for clinical applications to a wide range of CAR-T-cell therapies against solid tumours. First, collagen is abundantly expressed across patients with multiple tumour types, probably making this technology useful for a wide variety of patients. Second, identifying a promising target antigen for each solid tumour type is a major challenge in the field^4,5^, while our versatile, collagen-targeting approach enables a payload to avoid competition with CARs. Third, CBD is unlikely to confer immunogenicity to CAR-T cells because it is of fully human origin (the anti-CBD-IL-12 IgG induction observed in the RM9-hSTEAP1 syngeneic mouse model is less likely to happen in humans).

In summary, this study demonstrates a protein-engineering approach to make IL-12 safer for use with CAR-T therapy. The use of CBD-IL-12 lies in its capacity to overcome the immunosuppressive TME and antigen heterogeneity while avoiding irAEs and competition with CARs for tumour targeting. CBD-hIL-12-armoured human CAR-T cells showed potent anti-tumour efficacy while minimizing IL-12 in the circulation in 22Rv1 human prostate cancer, demonstrating a high potential for clinical translation.

## Methods

### Cell lines

HEK293T (CRL-3216), RM9 (CRL-3312), MyC-CaP (CRL-3255), 22Rv1 (CRL-2505) and Jurkat (TIB-152) cells were obtained from ATCC. HEK293T, RM9 and MyC-CaP cells were maintained in DMEM medium (Gibco) supplemented with 10% FBS (Gibco), 2 mM GlutaMAX (Gibco) and 1% penicillin/streptomycin (Gibco). 22Rv1 and Jurkat cells were maintained in RPMI 1640 medium (Gibco) supplemented with 10% FBS, 2 mM GlutaMAX and 1% penicillin/streptomycin. PLAT-E (RV-101) was obtained from Cell Biolabs and maintained in DMEM medium supplemented with 10% FBS, 2 mM GlutaMAX, 1% penicillin/streptomycin, 1 µg ml^−1^ puromycin (Merck) and 10 µg ml^−1^ blasticidin (Stratech). RM9-hSTEAP1-Fluc, 22Rv1-Fluc-RFP, 22Rv1 hSTEAP1 KO and 22Rv1 hSTEAP1 KO + rescue cells were generated in our previous work^24^. Myc-CaP-hSTEAP1 cells were generated using a FU-hSTEAP1-CGW vector^24^.

### Validation of hSTEAP1 expression on prostate cancer cells

Cells were resuspended in cold phosphate-buffered saline without calcium and magnesium (PBS (−)) and stained with BD Horizon Fixable Viability Stain 510 (BD). After a wash with PBS (−) supplemented with 2% FBS, the cells were stained with Vandortuzumab (Invitrogen, 1:200), followed by Alexa Fluor 594 AffiniPure F(ab’)_2_ Fragment Donkey anti-human IgG (H + L) (Jackson ImmunoResearch, 709-586-149, 1:200). Cells were acquired using a BD FACSymphony A3 flow cytometer and data were analysed using FlowJo (BD).

### Mice

All animals used in Imperial College London were handled in accordance with the 1986 Animal Scientific Procedures Act and under a United Kingdom Government Home Office-approved project licence and overseen by ethics committees of Imperial College London. Male C57BL/6J (6–9 weeks), FVB/N mice (6–9 weeks) and NSG (NOD-SCID-IL2Rγ-null) mice (3–5 weeks) were obtained from Charles River UK and housed at the Hammersmith Central Biomedical Services facility of Imperial College London. A 12-h light/dark cycle was used, room temperature was maintained between 20 and 21 °C, and room humidity was maintained at 50 ± 10%. All mouse studies performed in UCLA were in accordance with protocols approved by the UCLA Institutional Animal Care and Use Committee, known as the Chancellor’s Animal Research Committee (ARC). Male NSG mice (4–6 weeks) were obtained from The Jackson Laboratory and housed in UCLA, California NanoSystems Institute (CNSI) animal housing facility. A 12-h light/dark cycle was used, room temperature was maintained at 23 ± 3 °C, and room humidity was maintained at 50 ± 20%. BLI was performed using the IVIS Spectrum (PerkinElmer) at the Preclinical Imaging Technology Center at the Crump Institute for Molecular Imaging, CNSI.

### Production and purification of recombinant scIL-12 variants

Optimized sequences encoding p35 and p40 connected with a glycine-serine linker were synthesized and subcloned into pcDNA3.1(+) vector by GenScript. The collagen-binding domain (A3 domain of human vWF)^47^ was fused to the N terminus, C terminus or both termini of the protein to create CBD-IL-12 variants. His-tag was added to the C terminus after the CBD. Recombinant IL-12 variants were produced by transient expression in HEK293F cells and purified as described previously^22^. The purity of the proteins was evaluated using SDS–PAGE as described previously^22^. Protein concentration was quantified by measuring absorbance at 280 nm using a NanoDrop One spectrophotometer (Thermo Fisher). Full amino acid sequences of the IL-12 variants are available in the Supplementary Information.

### STAT4 phosphorylation assay

Primary mouse T cells were treated with IL-12 variants to evaluate phosphorylation of STAT4 as described previously^22^. In brief, CD3^+^ T cells were purified from spleens of C57BL/6J mice using the MagniSort Mouse T cell Enrichment kit (Invitrogen) according to manufacturer instructions. Purified T cells were activated using the T cell Activation/Expansion kit, mouse (Miltenyi Biotec) according to manufacturer instructions and cultured in the mouse T cell culture medium (RPMI 1640 medium supplemented with 10% FBS, 2 mM GlutaMAX, 1% MEM non-essential amino acids (Gibco), 1 mM sodium pyruvate (Gibco), 50 µM 2-mercaptoethanol (Gibco), 1% penicillin/streptomycin and 10 ng ml^−1^ recombinant human IL-2 (Peprotech)) for 3 days. T cell activation/expansion beads were magnetically removed, and the T cells were rested in fresh culture medium (without IL-2) overnight. T cells were seeded into 96-well V-bottom plates at 1 × 10^5^ cells per well; stimulated with the indicated concentrations of IL-12 variants at 37 °C for 15 min; fixed with BD Phosflow Lyse/Fix buffer and permeabilized with BD Phosflow Perm Buffer III according to manufacturer instructions; stained with anti-pSTAT4 Alexa Fluor 647 (pY693, BD, 1:100) and anti-mouse CD8α BV510 (53-6.7, Biolegend, 1:200); and then acquired using a BD FACSymphony A3 Cell Analyzer.

### CAR expression plasmids

pSIRV-NFAT-eGFP was a gift from Peter Steinberger (Addgene, plasmid 118031). pHR_SFFV was a gift from Wendell Lim (Addgene, plasmid 79121). hSTEAP1-BBζ CAR sequences were prepared as described previously^24^. SFFV promoter, hSTEAP1-mBBζ CAR, mouse scIL-12 variant and WPRE were subcloned into the pSIRV-NFAT-eGFP vector via in-fusion cloning (Takara Bio) as illustrated in Fig. 1d. The eGFP sequence was replaced with the IL-12 sequence during the process. MSCV promoter, NFAT-responsive promoter, human scIL-12 variant, WPRE and hSTEAP1-hBBζ CAR sequences were cloned into pRRLSIN vector backbone^48^.

### Production of gamma-retroviral vectors

A day before transfection, PLAT-E cells were resuspended in antibiotic-free medium with 10% FBS and GlutaMAX, seeded and cultured for 24 h. PLAT-E cells were transfected with a retroviral transfer vector using GeneJuice (Merck Millipore). Supernatant was collected at 48 h and 72 h after transfection and passed through a 0.45 µm PES filter (Merck). Supernatant collected at 48 h was stored at 4 °C overnight and pooled with supernatant collected at 72 h. Pooled supernatant was concentrated by high-speed centrifugation (24,000 *g* at 4 °C for 2 h) using an Avanti JXN-30 centrifuge (Beckman Coulter) with a JS-24.15 rotor (Beckman Coulter) and resuspended in DMEM medium supplemented with 10% FBS^49^. Concentrated viral vector was immediately added to a non-treated cell culture plate pre-coated with 20 µg ml^−1^ RetroNectin (Takara Bio) and centrifuged (2,000 *g* at 32 °C for 1.5 h) before transduction of T cells.

### Producion of lentiviral vectors

psPAX2 (Addgene, plasmid 12260) was a gift from Didier Trono. HEK293T cells (ATCC) maintained in DMEM medium (Gibco) supplemented with 10% FBS (Gibco), 2 mM GlutaMAX (Gibco) and 1% penicillin/streptomycin (Gibco) were used. HEK293T cells were transfected with a lentiviral transfer vector, psPAX2 and pVSVg^24^.

### Manufacture of primary mouse CAR-T cells

Splenocytes were collected from spleens of male C57BL/6J or FVB/N mice (Charles River UK). A spleen was put on a 70-µm cell strainer pre-wet with DMEM supplemented with 2% FBS and mashed using the plunger of a 2.5-ml syringe (Terumo). The strainer was washed with DMEM medium twice and centrifuged at 300 *g* for 5 min. After removing the supernatant, red blood cells were lysed using ACK lysing buffer (Gibco) at room temperature for 5 min. Cells were washed with excess PBS (−) and resuspended in PBS (−) supplemented with 2% FBS and 2 mM EDTA. Mouse T cells were purified as described above. Purified T cells were activated using T cell Activation/Expansion kit, mouse, and cultured in the mouse T cell culture medium as described above. After 24 h, the activation/expansion beads were magnetically removed and 1 µg ml^−1^ anti-mouse IL-12 p40 (C17.8, Bio X Cell) was added for IL-12 CAR-T cells and CBD-IL-12 CAR-T cells. T cells were seeded into a RetroNectin-treated viral vector-coated plate, centrifuged (300 *g* at 32 °C for 10 min) and cultured for 48 h. After 48 h of culture, the cytokine supplementation was changed from recombinant human IL-2 to 10 ng ml^−1^ recombinant human IL-7 (Peprotech) and 10 ng ml^−1^ recombinant human IL-15 (Peprotech)^49^. %CAR^+^ was assessed using either a combination of biotinylated recombinant protein L (Thermo Scientific, 1:3,000) and streptavidin-Alexa Fluor 647 conjugate (Invitrogen, 1:1000) (for Fig. 2a–i) or a combination of Biotin-SP-conjugated AffiniPure F(ab’)_2_ Fragment Goat Anti-Human IgG, F(ab’)_2_ Fragment Specific (Jackson ImmunoResearch, 109-066-006, 1:200) and phycoerythrin (PE)-streptavidin (Biolegend, 1:200) (for all other figures) on day 4 by flow cytometry. CAR-T cells were resuspended in cold PBS (−), stained with acridine orange/propidium iodide (DeNovix) and counted using a CellDrop automated cell counter (DeNovix) to adjust the concentration before use in experiments. CAR-T cells were intravenously injected to mice on day 5 after viral transduction.

### CAR-T-cell-mediated cytotoxicity assay (calcein-AM)

CAR-T cells (1 × 10^5^ per ml) in the culture medium without supplementation of any interleukins were seeded into a 96-well V-bottom plate. Target cells were labelled with 1 µM calcein-AM as described previously^50^. Labelled target cells (1 × 10^5^ per ml) in the culture medium with 5 mM probenecid were mixed with the CAR-T cells at 10,000 target cells per well. The cells were centrifuged (300 *g* for 5 min at 37 °C) and incubated for 24 h at 37 °C in the presence of 5% CO_2_. The plate was centrifuged and 100 µl per well of the supernatant was transferred to a 96-well black F-bottom plate (Greiner Bio-One). Fluorescence intensity (Ex 490 nm, Em 530 nm) was measured using a CLARIOstar Plus microplate reader (BMG Labtech). After subtracting the autofluorescence derived from medium, % lysis was calculated as:

1 $$\% {\rm{lysis}}=100\times ({{F}}_{1}-{{F}}_{{\rm{E}}}-{{F}}_{0})/({{F}}_{100}-{{F}}_{0})$$

*F*_1_: Fluorescence intensity of the mixture of CAR-T cells and labelled target cells

*F*_E_: Fluorescence intensity of CAR-T cells

*F*_0_: Fluorescence intensity of labelled target cells

*F*_100_: Fluorescence intensity of labelled target cells lysed with 1% Triton X-100

### Enzyme-linked immunosorbent assay (ELISA) for detection of IL-12 and IFNγ secreted from CAR-T cells

CAR-T cells (5 × 10^5^ per ml) in the culture medium (without supplementation of any interleukins) and 5 × 10^5^ target cells per ml in the same medium were seeded into a 96-well V-bottom plate (50,000 CAR-T cells per well, CAR-T/Target ratio = 1), centrifuged (300 *g* for 5 min at 37 °C) and incubated for 24 h at 37 °C in the presence of 5% CO_2_. The plate was centrifuged again and the culture supernatant was collected for quantification of IL-12 and IFNγ by ELISA. Invitrogen ELISA kits were used for mouse proteins. Human IL-12 was quantified using IL-12 p70 DuoSet (R&D Systems). eBioscience Cell Stimulation Cocktail (Invitrogen) was used as a cocktail of phorbol 12-myristate 13-acetate (PMA) and ionomycin. For quantification of scIL-12 variants secreted from CAR-T cells, the IL-12 variants with His-tag were used as standards.

### Intracellular IL-12 staining

Primary mouse T cells were stimulated with a cocktail of PMA and ionomycin in the presence of 5 µg ml^−1^ Brefeldin A overnight. Cells were fixed and permeabilized using the eBioscience intracellular fixation and permeabilization buffer (Invitrogen) and stained with rat anti-mouse IL-12 PE (C15.6, BD, 1:100).

### RT–qPCR of mouse IL-12 transcripts

CAR-T cells (1 × 10^6^ per sample) were stimulated with PMA and ionomycin for 4 h. Total RNA was purified using RNeasy Mini kit (Qiagen) according to manufacturer instructions, quantified using Nanodrop One and stored at −80 °C until use. Reverse transcription was performed using *Taq*Man Reverse Transcription Reagents (Invitrogen) using random hexamers. qPCR was performed using SYBR Green Universal Master Mix. Expression levels of mouse p35 and p40 in each sample were normalized by an internal control (EGFP for EGFP + NFAT-CBD-IL-12-CBD; WPRE for hSTEAP1-mBBζ + NFAT-CBD-IL-12 and hSTEAP1-mBBζ + NFAT-IL-12-CBD) and relative gene expression changes with PMA and ionomycin stimulation were calculated. The following primers were used (primer sequences for IL-12 subunits are optimized for the gamma-retroviral vectors used. Thus, their sequences are not completely matched to mouse genomic sequence): WPRE F: 5’-GTGGATACGCTGCTTTAATGCCT-3’, WPRE R: 5’-GTTGCGTCAGCAAACACAGT-3’; EGFP F: 5’-AAGGGCATCGACTTCAAGG-3’, EGFP R: 5’-TGCTTGTCGGCCATGATATAG-3’; p40 F: 5’-TCGAGCTGGCCCTGGAGG-3’, p40 R: 5’-CGACCTGGCTGTTCTTCAGGG-3’; p35 F: 5’-ACGAGTCTTGTCTGGCCACC-3’, p35 R: 5’-CCTCGTAGATGCTGCCCAGG-3’.

### Anti-tumour efficacy of CAR-T cells against RM9-hSTEAP1

A total of 5 × 10^5^ RM9-hSTEAP1-Fluc cells resuspended in 50 µl of PBS (−) were subcutaneously injected on the left side of the back of each male C57BL/6J mouse on day 0. Primary mouse CAR-T cells were intravenously injected on day 4 or day 6. The dose of CAR-T cells in each study is described in figure legends. Heterodimeric CBD-IL-12 protein^22^ (10 µg or 25 µg) was intravenously injected on day 8 in Fig. 2n–p. In some studies, anti-CTLA-4 (9H10, Bio X cell) and anti-PD-1 (RMP1-14, Bio X cell) were intraperitoneally injected (100 µg each) 3 times starting at 3 days after the CAR-T administration with 4 days interval. Tumours were measured using a digital caliper and volumes were calculated as ellipsoids (*V* = 4/3 × 3.14 × depth/2 × width/2 × height/2). Blood samplings from tail veins were performed at the indicated timepoints to assess serum concentration of IFNγ by ELISA. Serum samples that showed values below the detection limit were plotted at the detection limit value in the logarithmic graph. In some studies, complete responders received subcutaneous tumour rechallenge (5 × 10^5^ cells) as indicated in experimental timelines and figure legends. Mice were euthanized when tumour volume had exceeded 500 mm^3^, or tumour ulceration of more than 5 mm in diameter had been observed.

### Detection of serum anti-IL-12 IgG

Blood samples were collected in K3 EDTA-coated tubes (Sarstedt). The samples were centrifuged at 2,000 *g* for 10 min at 4 °C. Plasma was collected in protein lobind tubes (Eppendorf) and stored at −20 °C until use. A 96-well F-bottom plate (medium binding, Greiner Bio-One) was coated with 5 µg ml^−1^ of mouse scIL-12-His or molar equivalent of CBD-mouse scIL-12-His in PBS overnight at 37 °C. Following three washes with PBS-T (0.05% Tween 20), the plate was blocked with 2% BSA in PBS-T for 1 h at room temperature. Plasma samples diluted 10× in PBS (−) were added and incubated for 2 h at room temperature. After three washes, Peroxidase AffiniPure F(ab’)_2_ Fragment Goat Anti-Mouse IgG (H + L) (Jackson ImmunoResearch, 115-036-003) diluted in PBS (1:10,000) was added and incubated for 1 h at room temperature. After four washes, eBioscience TMB solution (Invitrogen) was added, followed by 0.13 M H_2_SO_4_ to stop the reaction. The values obtained by subtracting the absorbance at 620 nm from the absorbance at 450 nm are shown.

### Quantification of IL-12 in serum, tumour and major organs

Tumour-bearing mice received intravenous injection of CAR-T cells as indicated in experimental timelines and figure legends. Blood samples, tumours and major organs were collected at the indicated timepoints. Serum was collected in 1.5 ml protein lobind tubes (Eppendorf) and stored at −80 °C until use. Parts of the removed tumours and organs were immediately put into pre-weighed Lysing Matrix D tube (MP Biomedicals) containing 1 ml of T-PER Tissue Protein Extraction reagent (Thermo Scientific) supplemented with cOmplete EDTA-free protease inhibitor cocktail (Roche). Tubes with tissue samples were weighed again and tissues were cut into small pieces using surgical scissors. The samples were lysed using FastPrep-24 5G (MP Biomedicals) and stored at −80 °C until use. IL-12 was quantified using mouse IL-12 p70 ELISA kit (Invitrogen) or Human IL-12 p70 DuoSet ELISA (R&D Systems) and normalized to total tissue weight. Recombinant CBD-IL-12 and IL-12 (Supplementary Table 1) produced in-house were used as standards. Tumour and organs of RM9-hSTEAP1-bearing mice receiving 10 million UTD T cells were used to obtain background IL-12 signals for Fig. 3a–h.

A total of 2 × 10^6^ MyC-CaP-hSTEAP1 cells resuspended in 50 µl of 50% PBS (−) and 50% Phenol Red-free Matrigel (Corning) were subcutaneously injected on the left side of the back of each male FVB/N mouse. MyC-CaP-hSTEAP1 tumour-bearing FVB/N mice received 5 million CAR-T cells when average tumour volume was ~230 mm^3^. Tumours were collected 4 days after CAR-T cell administration and 3 fragments were obtained from each tumour tissue for the experiment. Intratumoural IL-12 was quantified as described above and normalized to total protein content. Total protein content was quantified using the Pierce BCA Protein Assay kit (Thermo Fisher). Bovine serum albumin was used as standard.

Subcutaneous MyC-CaP-hSTEAP1 tumours were established in male NSG mice as described above. Five million CAR-T cells (derived from male C57BL/6J mice) were intravenously injected on day 8. Blood was collected from the tail vein on day 12 for serum IL-12 quantification. The study was terminated on day 16 due to rapid body weight loss indicating graft-versus-host disease.

### Systemic toxicity of armoured CAR-T-cell therapy

RM9-hSTEAP1-Fluc tumour-bearing mice received 10 million CAR-T cells on day 4 after tumour inoculation. Terminal blood sampling by cardiac puncture for blood chemistry analysis was performed on day 12. Serum ALT and ALP were quantified using the Skyla VB1 veterinary chemistry analyser. Skyla pre-anaesthetic panel discs were used according to manufacturer instructions.

### Quantification of cytokines in CAR-T-treated tumours

RM9-hSTEAP1-Fluc tumour-bearing mice received 10 million CAR-T cells on day 4 after tumour inoculation. Tumours were collected on day 12 and lysed as described above. All samples were stored at −80 °C until use. Intratumoural cytokines were quantified using a LEGENDplex kit (Biolegend) according to manufacturer instructions and normalized to total protein content.

### Histological analysis of immune infiltrates in tumour, lung, liver and kidney

RM9-hSTEAP1-Fluc tumour-bearing B6 mice received 5 million CAR-T cells on day 4 after tumour inoculation. Tumours, lungs, livers and kidneys were collected on day 14 and fixed with 4% paraformaldehyde solution in PBS overnight at 4 °C. Formalin-fixed, paraffin-embedded tissue samples were sectioned, followed by H&E staining and immunohistochemistry analysis as previously described^24^. Briefly, tissue slides were deparaffinized and rehydrated, followed by antigen retrieval in Citrate-Based Antigen Unmasking Solution (Vector Labs). Slides were blocked and stained with rabbit anti-CD3 antibody (Thermo Fisher, MA5-14524, 1:100) for 1 h at 37 °C. Slides were washed three times with TBST (pH 8.0) and incubated with PowerVision Poly-HRP anti-rabbit IgG (Leica Biosystems, 1:100) at 37 °C for 30 min. After three washes, slides were incubated with 3,3′-diaminobenzidine (DAB) (Sigma Aldrich) at room temperature for 10 min. Slides were stained for H&E, followed by dehydration steps and mounting. Stained slides were scanned at the Translational Pathology Core Laboratory (TPCL) at UCLA. QuPath 0.5.1 was used to quantify CD3^+^ area in the IHC images^51^. The average channel was used for tissue detection, followed by the DAB channel for detection of CD3^+^ stained area.

### Analysis of immune cells in tumour using flow cytometry

RM9-hSTEAP1-Fluc tumour-bearing mice received 5 million CAR-T cells on day 4 after tumour inoculation. Tumours were collected on day 14 and cut into small pieces using surgical scissors and digested in DMEM medium supplemented with 2% FBS, 2 mg ml^−1^ collagenase D (Sigma Aldrich) and 40 µg ml^−1^ DNase I (Roche) for 30 min at 37 °C. Single-cell suspensions in DMEM supplemented with 2% FBS were prepared from digested tumours using a 70-µm cell strainer (Thermo Fisher). Red blood cells were lysed with ACK lysing buffer (Gibco) for 5 min at room temperature and neutralized with PBS (−). Cells were stained with BD Horizon Fixable Viability Stain 510 (BD). Fc receptors were blocked using purified anti-mouse CD16/32 antibody (93, Biolegend, 1:200). Cells were stained with a cocktail of anti-mouse antibodies, fixed with eBioscience IC Fixation Buffer (Invitrogen) and acquired using a BD FACSymphony A3 flow cytometer, and data were analysed using FlowJo (BD). Simple linear regression was performed to analyse the correlation between tumour burden and immune cell infiltrates using GraphPad Prism 10. The following anti-mouse antibodies were used: CD45.2 APC-Cy7 (30-F11, Biolegend, 1:200), CD3 BUV395 (145-2C11, BD, 1:100), CD4 BUV805 (GK1.5, BD, 1:200), CD8 Alexa Fluor 700 (53-6.7, Biolegend, 1:200), NK1.1 PerCP-Cy5.5 (PK136, Biolegend, 1:200), PD-1 BV605 (29F.1A12, Biolegend, 1:200), CTLA-4 APC (UC10-4B9, Biolegend, 1:200), CD11b APC-Cy7 (M1/70, Invitrogen, 1:200), Ly6G BUV737 (1A8, BD, 1:200), Ly6C Alexa Fluor 488 (HK1.4, Biolegend, 1:200), CD19 BV785 (6D5, Biolegend, 1:200), CD11c PE-Cy7 (HL3, BD, 1:200), MHC-II (I-A/I-E) BV711 (M5/114.15.2, Biolegend, 1:200) and CD103 BV605 (2E7, Biolegend, 1:200). Gating strategies are shown in Supplementary Figs. 5 and 6.

### GeoMx High-plex transcriptome analysis

NanoString GeoMx Digital Spatial Profiler (DSP) whole transcriptome analysis (WTA) was performed on tumour sample collected at day 14 after tumour inoculation. Parafin-embedded tissue blocks were sectioned by the UCLA Translational Pathology Core Laboratory (TPCL). NanoString GeoMx DSP was performed at the Technology Center for Genomics and Bioinformatics (TCGB) core at UCLA. Twelve regions of interest (ROI) were chosen across four different tumour sections in each treated group, followed by library preparation and sequencing. A tumour sample in the unarmoured CAR-T treatment group was not used because the tissue section was damaged.

Transcriptome data analysis was performed using NanoString Spatial Data Analysis software (GeoMx DSP Software v.3.0.0.109). Raw files were analysed for quality control, followed by sequence alignment. Count matrix files were used to perform differential gene expression analysis using DESeq2 (ref. ^52^). For PCA analysis, fragments per kilobase of transcript per million mapped reads values were normalized by log_2_ + 1 transformation and PCA was plotted on the basis of a correlation matrix using the prcomp package v.3.6.2. PCA plots were visualized using the factoextra package v.1.0.7 and ggpubr package v.0.6.0. S1-12 was excluded as an outlier in GSEA on the basis of the PCA analysis. Pathway analysis was performed using GSEA (v.4.3.3)^53^. All computational analyses were carried out in RStudio v.4.1.0. Heat maps were generated using the package pheatmap v.1.0.12. One-sided Fisher’s exact test was performed using the fisher.test() function in R.

### Splenic T cell response to hSTEAP1-positive and negative RM9 cells after CAR-T treatment

RM9-hSTEAP1-Fluc tumour-bearing mice received 10 million CAR-T cells on day 4 after tumour inoculation. Spleens were collected on day 12 and splenic T cells were purified. The T cell enrichment cocktail was supplemented with 1 µg ml^−1^ Biotin-SP (long spacer) AffiniPure F(ab’)_2_ Fragment Donkey Anti-Human IgG (H + L) (Jackson ImmunoResearch, 709-066-149) to remove CAR-T cells. Isolated splenic T cells were maintained in the mouse T cell culture medium until use. A day before co-culture with splenic T cells, 3 × 10^6^ tumour cells were seeded in a T75 flask, supplemented with 20 ng ml^−1^ recombinant mouse IFNγ (Biolegend) and incubated for 24 h at 37 °C in the presence of 5% CO_2_. On the next day, tumour cells were collected using TryPLE express Enzyme (Gibco) and washed twice with the mouse T cell culture medium without cytokines. Tumour cells (2 × 10^5^) and the same number of splenic T cells were mixed in 200 µl of mouse T cell culture medium without cytokines, seeded in a 96-well V-bottom plate (Corning) and incubated for 48 h at 37 °C in the presence of 5% CO_2_. Supernatant was collected and mouse IFNγ was quantified by ELISA.

### Manufacture and functional testing of CAR Jurkat cells

Jurkat cells were transduced with 10× concentrated lentiviral vector using a Retronectin-coated plate as described above. Surface expression of the CAR was detected using Biotin-SP-conjugated AffiniPure F(ab’)2 Fragment Goat Anti-Human IgG, F(ab’)2 Fragment Specific (Jackson ImmunoResearch, 109-066-006, 1:200) and PE-streptavidin (Biolegend, 1:200). CAR^+^ Jurkat T cells (50,000) were stimulated with eBioscience cell stimulation cocktail (Invitrogen). Secreted IL-12 variants were quantified by ELISA. Human scIL-12-His and CBD-human scIL-12-His were used as standards.

### Manufacture and functional testing of human CAR-T cells (A)

The following procedures were performed to generate data in Fig. 8a–c. Healthy donor human peripheral blood mononuclear cells (PBMCs) (STEMCELL Technologies) were subjected to EasySep CD8 negative selection (STEMCELL Technologies). CD8^+^ T cells (1 × 10^6^ per ml) were stimulated with T cell TransAct (1:100, Miltenyi Biotec) in RPMI media supplemented with 5% human serum (Bloodworks Northwest) and 50 U ml^−1^ human IL-2 (Peprotech). Next day, 5× concentrated lentiviral vector was added to the culture with 10 µg ml^−1^ protamine sulfate (MP Biomedicals). After 24 h, media were replaced with fresh IL-2 to remove viruses and TransAct. Seven days after the addition of viruses, cells were stained with biotin-anti-Fab (Jackson Immunoresearch, 109-066-006, 1:100) followed by PE-streptavidin (Thermo Fisher, 1:200), and CAR^+^ cells were sorted (except untransduced cells from which all live cells were sorted) on a BD FACSymphony S6 system. Sorted cells (5 × 10^4^) were expanded with irradiated feeder cells (2 × 10^6^ mixed PBMCs, three individual donor PBMCs from STEMCELL Technologies) and 4 × 10^5^ Epstein–Barr virus-transformed B-lymphoblastoid cells (Fred Hutch Research Cell Bank), 50 U ml^−1^ IL-2, 10 ng ml^−1^ IL-15 and 0.3 ml anti-CD3 antibodies (OKT3, Miltenyi Biotec) in 1 ml culture volume. After 10 days, T cells were used for functional assays. For the killing assay, 1 × 10^5^ T cells were co-cultured with 1 × 10^4^ target cells (22Rv1 hSTEAP1 KO + rescue or 22Rv1 hSTEAP1 KO)^24^ lentivirally transduced to express mCherry in 96-well flat-bottom plates (Corning). Target killing was monitored in IncuCyte (Sartorius), detecting fluorescence from mCherry every 4 h using whole-well scanning. The integrated intensity per well was normalized to values from the first timepoint. For cytokine production, 1 × 10^5^ each of T cells and target cells were co-cultured in 96-well round-bottom plates (Corning) with 1:1,000-diluted Golgi Stop (monensin, BD) and Golgi Plug (brefeldin A, BD) for 18 h. Cells were then surface stained with Live/Dead Aqua (Thermo Fisher, 1:1,000) and FITC-CD8 (SK1, Biolegend, 1:200) for 15 min at 4 °C, fixed and permeabilized with BD Cytofix/Cytoperm (BD), intracellularly stained with APC-IFNγ (4S.B3, Biolegend, 1:100) and PE-IL-12 (20C2, BD, 1:100) for 15 min at 4 °C, and analysed on a BD FACSymphony A5 system.

### Manufacture and functional testing of human CAR-T cells (B)

The following procedures were performed to generate data in Fig. 8d–h and Extended Data Fig. 10d–f. PBMCs were isolated from fresh whole human male blood in acid citrate dextrose (Research Donors) using Ficoll Paque Plus (Cytiva) and cryopreserved using CryoStor CS5 (STEMCELL Technologies) until use. CD3^+^ T cells were isolated using a Magnisort Human T cell Enrichment kit (Invitrogen) and activated using Dynabeads Human T-Activator CD3/CD28 (Thermo Fisher) in CTS OpTmizer T Cell Expansion SFM (Gibco) supplemented with CTS OpTmizer T Cell Expansion Supplement, 2 mM l-glutamine, 5% CTS Immune Cell SR (Gibco), 1% penicillin/streptomycin, 10 ng ml^−1^ recombinant human IL-2 and 0.5 ng ml^−1^ recombinant human IL-15. At 24 h after activation, T cells were transduced with a RetroNectin-coated plate as described above. After 2 days, the activation beads were magnetically removed, and T cells were expanded until use. %CAR^+^ was assessed using biotinylated anti-human IgG and streptavidin-PE as described above.

### Manufacture and functional testing of human CAR-T cells (C)

The following procedures were performed to generate data in Fig. 8i–l and Extended Data Fig. 10g–i. Human CAR-T cells were generated as previously described^24^. Briefly, PBMCs were isolated from a de-identified healthy donor. Cryopreserved PBMCs were thawed, washed and resuspended in T cell media (TCM base) consisting of AIM-V medium (Gibco) supplemented with 55 mM β-mercaptoethanol, human male AB plasma (Sigma) and GlutaMAX. Following cell counting, CD4^+^ and CD8^+^ T cells were isolated using human CD4 and CD8 microbeads (Miltenyi Biotec) according to manufacturer protocol. CD4^+^ T cells were cultured in CD4 media (TCM base supplemented with 0.5 ng ml^−1^ IL-15 and 5 ng ml^−1^ IL-7), and CD8^+^ T cells were cultured in CD8 media (TCM base supplemented with 50 U ml^−1^ IL-2 and 0.5 ng ml^−1^ IL-15). T cells were activated on Day 0 using Dynabeads Human T-Activator CD3/CD28 following manufacturer instructions. On Day 2, CD4^+^ and CD8^+^ T cells were transduced with high-titre lentivirus at a multiplicity of infection (MOI) of 12.5, based on titration on HEK293T cells. On Day 4, activation beads were removed, cells were counted, and transduction efficiency was assessed by flow cytometry using R-PE-conjugated Protein L (Sino Biological, 2 µl/2.5 × 10^5^ cells). CAR-modified T cells were expanded until day 14 before experiments. The CAR-T cells and 22Rv1-Fluc-RFP cells were co-cultured as reported previously to evaluate target cell killing and secretion of IL-12 and IFNγ^24^.

### In vivo functional assays of primary human CAR-T cells

For the subcutaneous 22Rv1 model, a total of 2 × 10^6^ 22Rv1-Fluc-RFP cells resuspended in 50 µl of 50% PBS (−) and 50% Phenol Red-free Matrigel were subcutaneously injected on the left side of the back of each male NSG mouse on day 0. The mice received 5 million CAR-T cells intravenously on day 13, followed by blood samplings on days 20, 24 and 28 for serum IL-12 quantification. Serum samples that showed values below the detection limit were plotted at the detection limit value in the logarithmic graph. Tumour burden was tracked using a digital caliper as described above. For the 22Rv1 disseminated tumour model, 1 × 10^6^ 22Rv1-Fluc-RFP cells were intravenously injected on day −14. Five million human UTD, hSTEAP1-BBζ and armoured hSTEAP1-BBζ CAR-T cells at a defined CD4:CD8 ratio of 1:1 suspended in 100 µl of PBS were injected intravenously on day 0. Serial BLI images were recorded to assess the progression of tumours, and retro-orbital bleeds were collected at day 12 to quantify serum IL-12.

### Statistical analysis

Data and statistical analyses were performed using MicroSoft Excel v.16.88 and GraphPad Prism 10 except for transcriptomics data, which were analysed using R (as described above). *P* < 0.05 was considered statistically significant. Tests used are indicated in figure legends.

### Reporting summary

Further information on research design is available in the Nature Portfolio Reporting Summary linked to this article.

## Supplementary information

Supplementary Information Supplementary Figs. 1–6.

Reporting Summary

Supplementary Data 1 Source Data Supplementary Figs. 3 and 4.

## Source data

Source Data Source Data for Figs. 1–5, 7 and 8 and Extended Data Figs. 2–4 and 6–10.

## Data Availability

Spatial transcriptomics data are available at the NCBI GEO repository under accession No. GSE300750 (ref. ^54^). Source data are provided with this paper.
