# Supplementary material for: Collagen-binding IL-12-armoured STEAP1 CAR-T cells reduce toxicity and treat prostate cancer in mouse models
Source: Nat Biomed Eng. 2025 Oct 23;10(4):630–46. doi: 10.1038/s41551-025-01508-3 (PMC13099423; doi:10.1038/s41551-025-01508-3)
Supplement: Supplementary file 1 — Supplementary Figs. 1–6. [file 41551_2025_1508_MOESM1_ESM.pdf]

nature biomedical engineering

Supplementary information https://doi.org/10.1038/s41551-025-01508-3

Collagen-binding IL-12-armoured STEAP1 CAR-T cells reduce toxicity and treat prostate cancer in mouse models

In the format provided by the authors and unedited

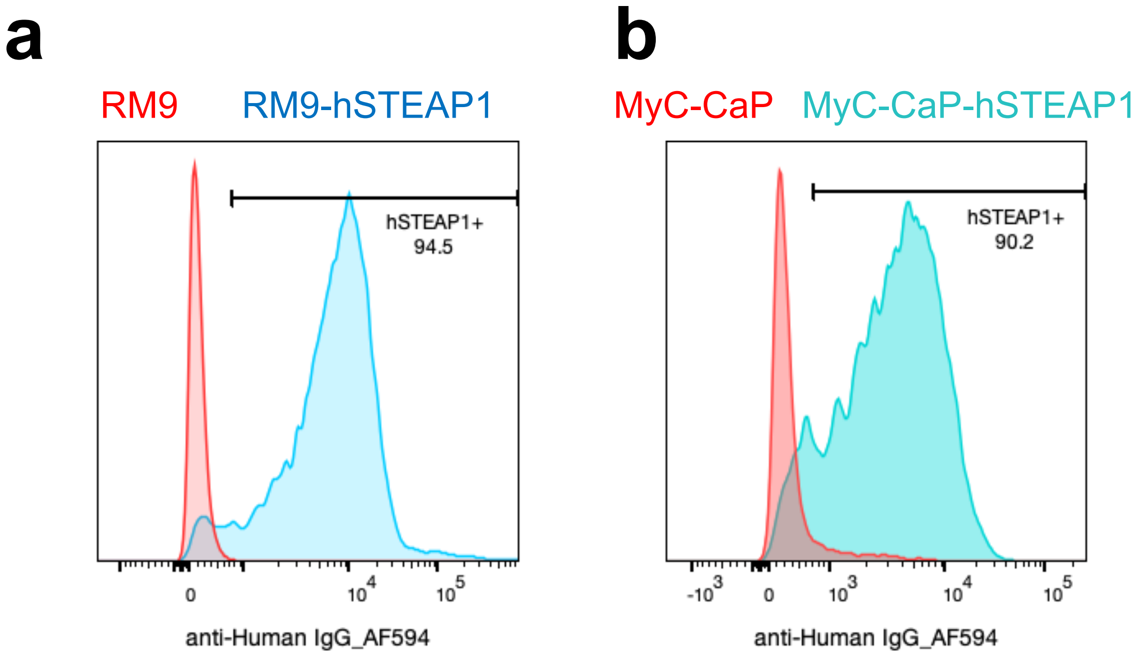

Supplementary Fig. 1 Flow cytometric analysis of human STEAP1 (hSTEAP1) expression in mouse prostate cancer cell lines.

(a) RM9 and (b) MyC-CaP cells were engineered to express hSTEAP1 by lentiviral transduction. The cells were stained with Vandortuzumab (anti-hSTEAP1 human IgG1) followed by anti-human IgG-Alexa Fluor 594. Representative histograms from an experiment.

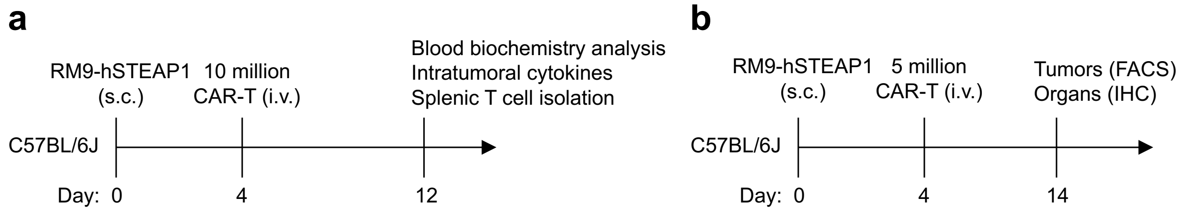

Supplementary Fig. 2 Experimental timelines, related to Figs. 4-6.

Male C57BL6/J mice received subcutaneous injection of RM9-hSTEAP1 (5×10^5) on day 0. (a) The tumor-bearing mice received 10 million CAR-T cells (i.v.) on day 4. Blood samples, tumors and spleens were collected for biochemistry analysis (Fig. 4a, b), intratumoral cytokine analysis (Fig. 5a-c) and splenic T cell restimulation (Fig. 5m). (b) The tumor-bearing mice received 5 million CAR-T cells (i.v.) on day 4. Organs (liver, kidney, lung) and tumors were harvested on day 14. The organs were processed for histological analysis (Fig. 4c-e, Extended Data Fig. 5). Tumors were used for flow cytometric analysis of immune infiltrates (Fig. 5e-l, Extended Data Fig. 6, Extended Data Fig. 7a, Extended Data Fig. 8) and spatial transcriptomics (Fig. 6, Extended Data Fig. 7).

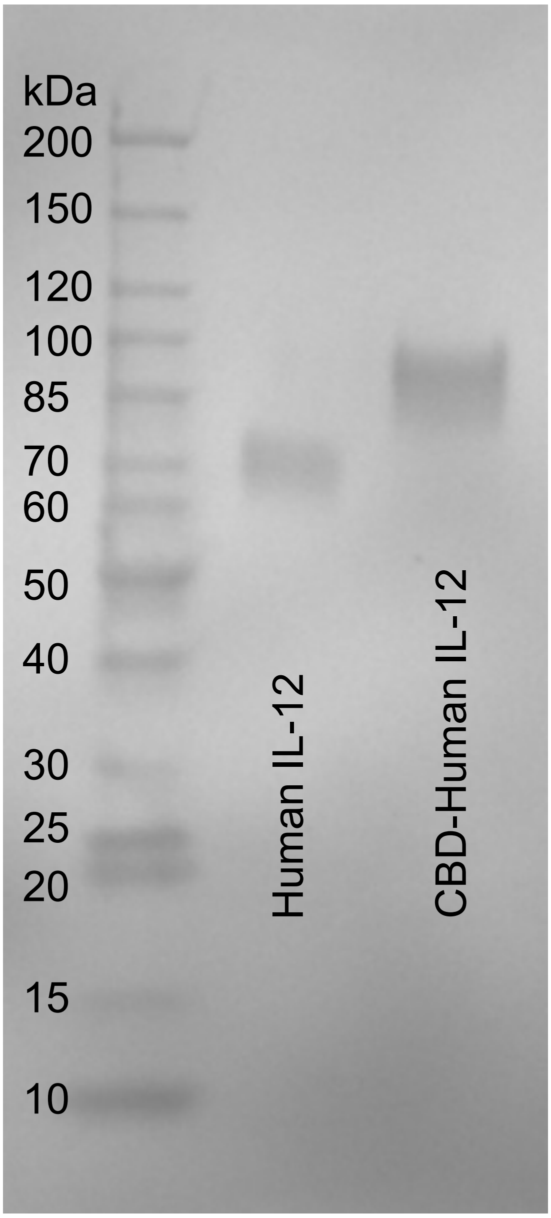

Supplementary Fig. 3 SDS-PAGE for recombinant human scIL-12 and CBD-human scIL-12.

Human scIL-12 variants were produced by transient expression in HEK293F cells, purified and analyzed by SDS-PAGE under reducing conditions with Coomassie blue staining.

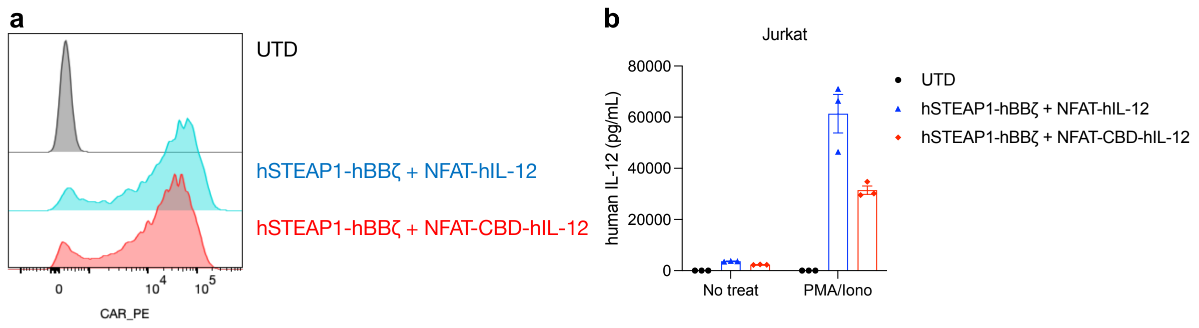

Supplementary Fig. 4 Jurkat T cells engineered to express hSTEAP1 CAR and CBD-human IL-12.

(a) Jurkat T cells were transduced to express the indicated transgenes. Cells were stained with Biotin-goat anti-Human IgG, and PE Streptavidin. Representative histograms of two experiments. (b) 50000 CAR$^+$ Jurkat T cells were stimulated with phorbol 12-myristate 13-acetate and ionomycin (PMA/Iono) for 24 h. Secreted IL-12 was quantified by ELISA (technical replicates, mean ± SEM). Human scIL-12-His and CBD-human scIL-12-His were used to draw standard curves.

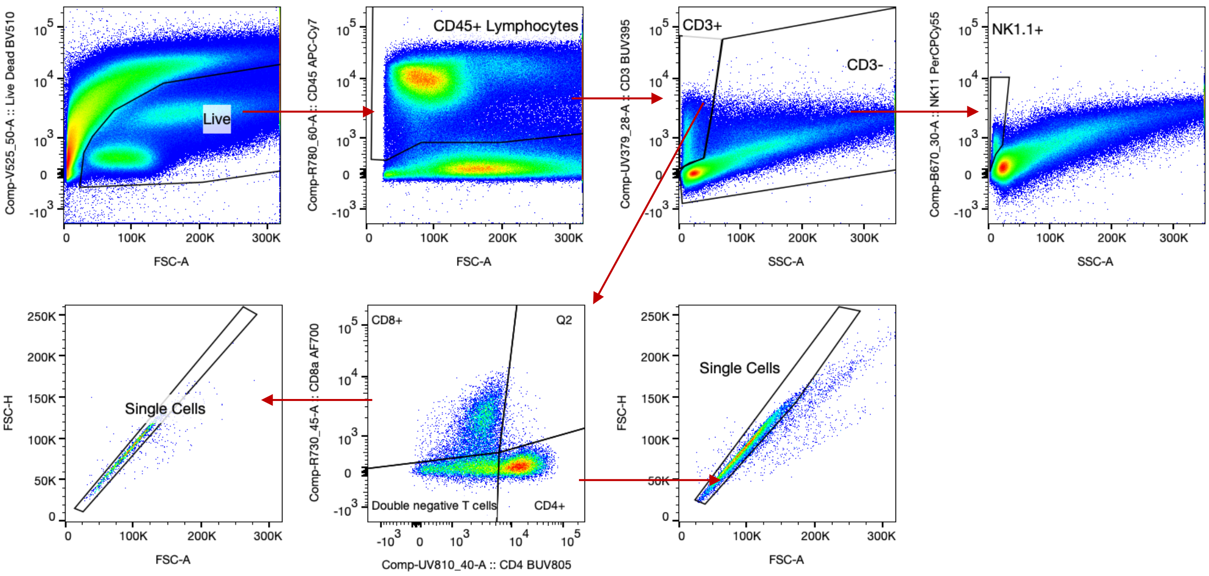

Supplementary Fig. 5 Gating strategy for characterizing T/NK cells.

Cells were first gated based on BD Horizon Fixable Viability Stain 510 followed by staining with indicated markers.

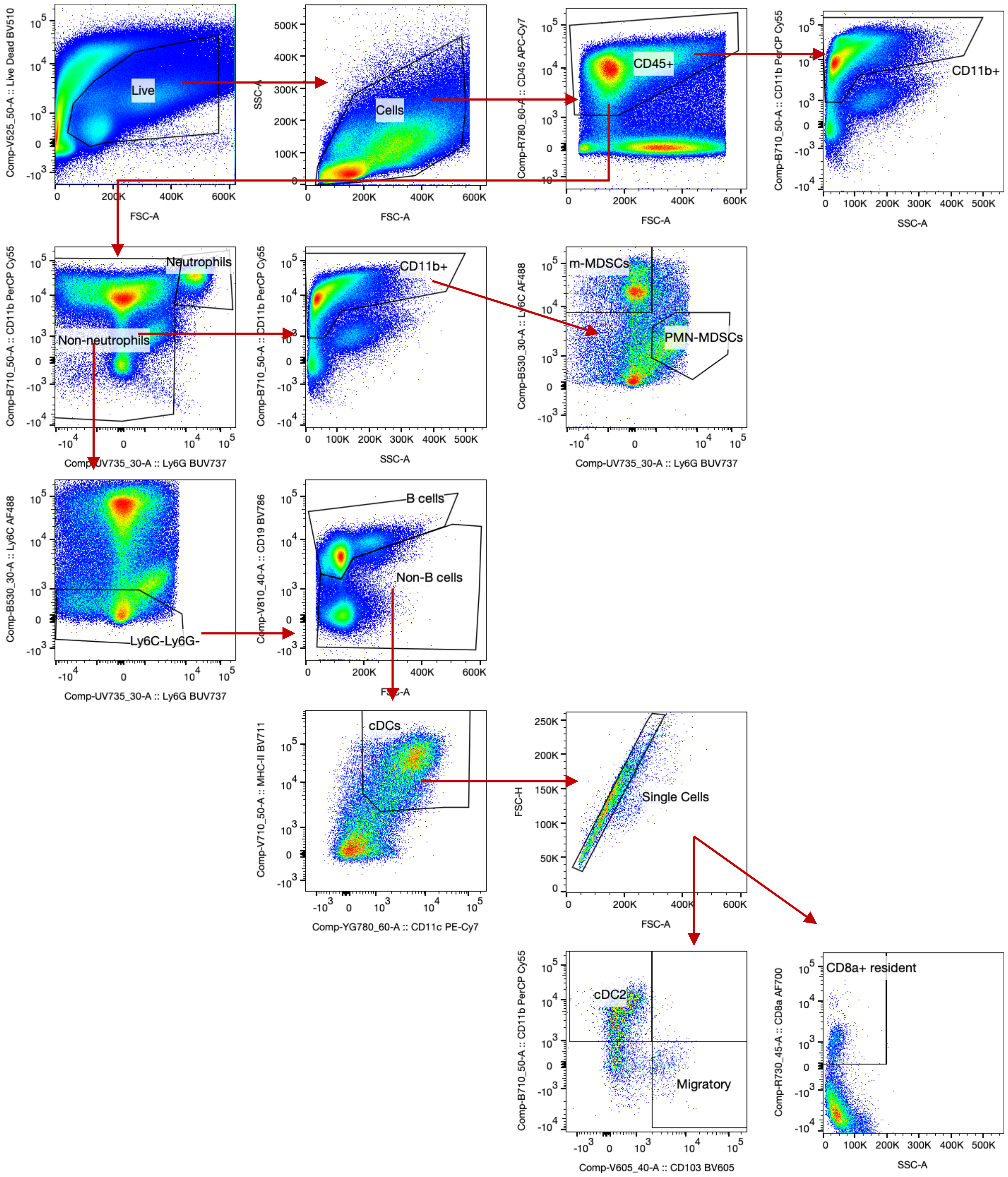

Supplementary Fig. 6 Gating strategy for characterizing B cells and myeloid cells.

Cells were first gated based on BD Horizon Fixable Viability Stain 510 followed by staining with indicated markers.

Supplementary Table 1. Amino acid sequences of the scIL-12 variants

Mouse IL-12 (for production in HEK293F):

MWELEKDVYVVEVDWTPDAPGETVNLTCDTPEEDDITWTSDQRHGVIGSGKTLTIT
VKEFLDAGQYTCHKGGETLSHSHLLLHKKENGIWSTEILKNFKNKTFLKCEAPNYS
GRFTCSWLVQRNMDLKFNIKSSSSSPDSRAVTCGMASLSAEKVTLDQRDYEKYSV
SCQEDVTCPTAEETLPIELALEARQQNKYENYSTSFFIRDIIKPDPPKNLQMKPLKNS
QVEVSWEYPDSWSTPHSYFSLKFFVRIQRKKEKMKETEEGCNQKGAFLVEKTSTE
VQCKGGNVCVQAQDRYYNSSCSKWACVPCRVRSGGSGGGSGGGSGGGSRVIPV
SGPARCLSQSRNLLKTTDDMVKTAREKLKHYSCTAEDIDHEDITRDQTSTLKTCLPL
ELHKNESCLATRETSSTTRGSCLPPQKTSLMMTLCLGSIYEDLKMYQTEFQAINAAL
QNHNHQQIILDKGMLVAIDELMQSLNHNGETLRQKPPVGEADPYRVKMKLCILLHAF
STRVVTINRVMGYLSSAHHHHHH*

Mouse IL-12 (as a payload for CAR-T):

MWELEKDVYVVEVDWTPDAPGETVNLTCDTPEEDDITWTSDQRHGVIGSGKTLTIT
VKEFLDAGQYTCHKGGETLSHSHLLLHKKENGIWSTEILKNFKNKTFLKCEAPNYS
GRFTCSWLVQRNMDLKFNIKSSSSSPDSRAVTCGMASLSAEKVTLDQRDYEKYSV
SCQEDVTCPTAEETLPIELALEARQQNKYENYSTSFFIRDIIKPDPPKNLQMKPLKNS
QVEVSWEYPDSWSTPHSYFSLKFFVRIQRKKEKMKETEEGCNQKGAFLVEKTSTE
VQCKGGNVCVQAQDRYYNSSCSKWACVPCRVRSGGSGGGSGGGSGGGSRVIPV
SGPARCLSQSRNLLKTTDDMVKTAREKLKHYSCTAEDIDHEDITRDQTSTLKTCLPL
ELHKNESCLATRETSSTTRGSCLPPQKTSLMMTLCLGSIYEDLKMYQTEFQAINAAL
QNHNHQQIILDKGMLVAIDELMQSLNHNGETLRQKPPVGEADPYRVKMKLCILLHAF
STRVVTINRVMGYLSSA*

CBD-mouse IL-12 (for production in HEK293F):

CSQPLDVILLLDGSSSFPASYFDEMKSFAKAFISKANIGPRLTQVSVLQYGSITTIDVP
WNVVPEKAHLLSLVDVMQREGGPSQIGDALGFAVRYLTSEMHGARPGASKAVVILV
TDVSVDSVDAAADAARSNRVTVFPIGIGDRYDAAQLRILAGPAGDSNVVKLQRIEDL
PTMVTLGNSFLHKLCSGFVRIGGGSGGGSMWELEKDVYVVEVDWTPDAPGETVN
LTCDTPEEDDITWTSDQRHGVIGSGKTLTITVKEFLDAGQYTCHKGGETLSHSHLLL
HKKENGIWSTEILKNFKNKTFLKCEAPNYSGRFTCSWLVQRNMDLKFNIKSSSSSP
DSRAVTCGMASLSAEKVTLDQRDYEKYSVSCQEDVTCPTAEETLPIELALEARQQN
KYENYSTSFFIRDIIKPDPPKNLQMKPLKNSQVEVSWEYPDSWSTPHSYFSLKFFVR
IQRKKEKMKETEEGCNQKGAFLVEKTSTEVQCKGGNVCVQAQDRYYNSSCSKWA
CVPCRVRSGGSGGGSGGGSGGGSRVIPVSGPARCLSQSRNLLKTTDDMVKTARE
KLKHYSCTAEDIDHEDITRDQTSTLKTCLPLELHKNESCLATRETSSTTRGSCLPPQ
KTSLMMTLCLGSIYEDLKMYQTEFQAINAALQNHNHQQIILDKGMLVAIDELMQSLN
HNGETLRQKPPVGEADPYRVKMKLCILLHAFSTRVVTINRVMGYLSSAHHHHHH*

CBD-mouse IL-12 (as a payload for CAR-T):

CSQPLDVILLLDGSSSFPASYFDEMKSFAKAFISKANIGPRLTQVSVLQYGSITTIDVP
WNVVPEKAHLLSLVDVMQREGGPSQIGDALGFAVRYLTSEMHGARPGASKAVVILV
TDVSVDSVDAAADAARSNRVTVFPIGIGDRYDAAQLRILAGPAGDSNVVKLQRIEDL
PTMVTLGNSFLHKLCSGFVRIGGGSGGGSMWELEKDVYVVEVDWTPDAPGETVN
LTCDTPEEDDITWTSDQRHGVIGSGKTLTITVKEFLDAGQYTCHKGGETLSHSHLLL
HKKENGIWSTEILKNFKNKTFLKCEAPNYSGRFTCSWLVQRNMDLKFNIKSSSSSP
DSRAVTCGMASLSAEKVTLDQRDYEKYSVSCQEDVTCPTAEETLPIELALEARQQN
KYENYSTSFFIRDIIKPDPPKNLQMKPLKNSQVEVSWEYPDSWSTPHSYFSLKFFVR
IQRKKEKMKETEEGCNQKGAFLVEKTSTEVQCKGGNVCVQAQDRYYNSSCSKWA
CVPCRVRSGGSGGGSGGGSGGGSRVIPVSGPARCLSQSRNLLKTTDDMVKTARE
KLKHYSCTAEDIDHEDITRDQTSTLKTCLPLELHKNESCLATRETSSTTRGSCLPPQ
KTSLMMTLCLGSIYEDLKMYQTEFQAINAALQNHNHQQIILDKGMLVAIDELMQSLN
HNGETLRQKPPVGEADPYRVKMKLCILLHAFSTRVVTINRVMGYLSSA*

Mouse IL-12-CBD (for production in HEK293F):

MWELEKDVYVVEVDWTPDAPGETVNLTCDTPEEDDITWTSDQRHGVIGSGKTLTIT
VKEFLDAGQYTCHKGGETLSHSHLLLHKKENGIWSTEILKNFKNKTFLKCEAPNYS
GRFTCSWLVQRNMDLKFNIKSSSSSPDSRAVTCGMASLSAEKVTLDQRDYEKYSV
SCQEDVTCPTAEETLPIELALEARQQNKYENYSTSFFIRDIIKPDPPKNLQMKPLKNS
QVEVSWEYPDSWSTPHSYFSLKFFVRIQRKKEKMKETEEGCNQKGAFLVEKTSTE
VQCKGGNVCVQAQDRYYNSSCSKWACVPCRVRSGGSGGGSGGGSGGGSRVIPV
SGPARCLSQSRNLLKTTDDMVKTAREKLKHYSCTAEDIDHEDITRDQTSTLKTCLPL
ELHKNESCLATRETSSTTRGSCLPPQKTSLMMTLCLGSIYEDLKMYQTEFQAINAAL
QNHNHQQIILDKGMLVAIDELMQSLNHNGETLRQKPPVGEADPYRVKMKLCILLHAF
STRVVTINRVMGYLSSACSQPLDVILLLDGSSSFPASYFDEMKSFAKAFISKANIGPR
LTQVSVLQYGSITTIDVPWNVVPEKAHLLSLVDVMQREGGPSQIGDALGFAVRYLTS
EMHGARPGASKAVVILVTDVSVDSVDAAADAARSNRVTVFPIGIGDRYDAAQLRILA
GPAGDSNVVKLQRIEDLPTMVTLGNSFLHKLCSGFVRIGGGSGGGSHHHHHH*

Mouse IL-12-CBD (as a payload for CAR-T):

MWELEKDVYVVEVDWTPDAPGETVNLTCDTPEEDDITWTSDQRHGVIGSGKTLTIT
VKEFLDAGQYTCHKGGETLSHSHLLLHKKENGIWSTEILKNFKNKTFLKCEAPNYS
GRFTCSWLVQRNMDLKFNIKSSSSSPDSRAVTCGMASLSAEKVTLDQRDYEKYSV
SCQEDVTCPTAEETLPIELALEARQQNKYENYSTSFFIRDIIKPDPPKNLQMKPLKNS
QVEVSWEYPDSWSTPHSYFSLKFFVRIQRKKEKMKETEEGCNQKGAFLVEKTSTE
VQCKGGNVCVQAQDRYYNSSCSKWACVPCRVRSGGSGGGSGGGSGGGSRVIPV
SGPARCLSQSRNLLKTTDDMVKTAREKLKHYSCTAEDIDHEDITRDQTSTLKTCLPL

ELHKNESCLATRETSSTTRGSCLPPQKTSLMMTLCLGSIYEDLKMYQTEFQAINAAL
QNHNHQQIILDKGMLVAIDELMQSLNHNGETLRQKPPVGEADPYRVKMKLCILLHAF
STRVVTINRVMGYLSSACSQPLDVILLLDGSSSFPASYFDEMKSFAKAFISKANIGPR
LTQVSVLQYGSITTIDVPWNVVPEKAHLLSLVDVMQREGGPSQIGDALGFAVRYLTS
EMHGARPGASKAVVILVTDVSVDSVDAAADAARSNRVTVFPIGIGDRYDAAQLRILA
GPAGDSNVVKLQRIEDLPTMVTLGNSFLHKLCSGFVRI*

CBD-mouse IL-12-CBD (for production in HEK293F):

CSQPLDVILLLDGSSSFPASYFDEMKSFAKAFISKANIGPRLTQVSVLQYGSITTIDVP
WNVVPEKAHLLSLVDVMQREGGPSQIGDALGFAVRYLTSEMHGARPGASKAVVILV
TDVSVDSVDAAADAARSNRVTVFPIGIGDRYDAAQLRILAGPAGDSNVVKLQRIEDL
PTMVTLGNSFLHKLCSGFVRIGGGSGGGSMWELEKDVYVVEVDWTPDAPGETVN
LTCDTPEEDDITWTSDQRHGVIGSGKTLTITVKEFLDAGQYTCHKGGETLSHSHLLL
HKKENGIWSTEILKNFKNKTFLKCEAPNYSGRFTCSWLVQRNMDLKFNIKSSSSSP
DSRAVTCGMASLSAEKVTLDQRDYEKYSVSCQEDVTCPTAEETLPIELALEARQQN
KYENYSTSFFIRDIIKPDPPKNLQMKPLKNSQVEVSWEYPDSWSTPHSYFSLKFFVR
IQRKKEKMKETEEGCNQKGAFLVEKTSTEVQCKGGNVCVQAQDRYYNSSCSKWA
CVPCRVRSGGSGGGSGGGSGGGSRVIPVSGPARCLSQSRNLLKTTDDMVKTARE
KLKHYSCTAEDIDHEDITRDQTSTLKTCLPLELHKNESCLATRETSSTTRGSCLPPQ
KTSLMMTLCLGSIYEDLKMYQTEFQAINAALQNHNHQQIILDKGMLVAIDELMQSLN
HNGETLRQKPPVGEADPYRVKMKLCILLHAFSTRVVTINRVMGYLSSACSQPLDVIL
LLDGSSSFPASYFDEMKSFAKAFISKANIGPRLTQVSVLQYGSITTIDVPWNVVPEKA
HLLSLVDVMQREGGPSQIGDALGFAVRYLTSEMHGARPGASKAVVILVTDVSVDSV
DAAADAARSNRVTVFPIGIGDRYDAAQLRILAGPAGDSNVVKLQRIEDLPTMVTLGN
SFLHKLCSGFVRIGGGSGGGSHHHHHH*

CBD-mouse IL-12-CBD (as a payload for CAR-T):

CSQPLDVILLLDGSSSFPASYFDEMKSFAKAFISKANIGPRLTQVSVLQYGSITTIDVP
WNVVPEKAHLLSLVDVMQREGGPSQIGDALGFAVRYLTSEMHGARPGASKAVVILV
TDVSVDSVDAAADAARSNRVTVFPIGIGDRYDAAQLRILAGPAGDSNVVKLQRIEDL
PTMVTLGNSFLHKLCSGFVRIGGGSGGGSMWELEKDVYVVEVDWTPDAPGETVN
LTCDTPEEDDITWTSDQRHGVIGSGKTLTITVKEFLDAGQYTCHKGGETLSHSHLLL
HKKENGIWSTEILKNFKNKTFLKCEAPNYSGRFTCSWLVQRNMDLKFNIKSSSSSP
DSRAVTCGMASLSAEKVTLDQRDYEKYSVSCQEDVTCPTAEETLPIELALEARQQN
KYENYSTSFFIRDIIKPDPPKNLQMKPLKNSQVEVSWEYPDSWSTPHSYFSLKFFVR
IQRKKEKMKETEEGCNQKGAFLVEKTSTEVQCKGGNVCVQAQDRYYNSSCSKWA
CVPCRVRSGGSGGGSGGGSGGGSRVIPVSGPARCLSQSRNLLKTTDDMVKTARE
KLKHYSCTAEDIDHEDITRDQTSTLKTCLPLELHKNESCLATRETSSTTRGSCLPPQ
KTSLMMTLCLGSIYEDLKMYQTEFQAINAALQNHNHQQIILDKGMLVAIDELMQSLN
HNGETLRQKPPVGEADPYRVKMKLCILLHAFSTRVVTINRVMGYLSSACSQPLDVIL

LLDGSSSFPASYFDEMKSFAKAFISKANIGPRLTQVSVLQYGSITTIDVPWNVVPEKA
HLLSLVDVMQREGGPSQIGDALGFAVRYLTSEMHGARPGASKAVVILVTDVSVDSV
DAAADAARSNRVTVFPIGIGDRYDAAQLRILAGPAGDSNVVKLQRIEDLPTMVTLGN
SFLHKLCSGFVRI*

Human IL-12 (for production in HEK293F):

IWELKKDVYVVELDWYPDAPGEMVVLTCDTPEEDGITWTLDQSSEVLGSGKTLTIQ
VKEFGDAGQYTCHKGGEVLSHSLLLLHKKEDGIWSTDILKDQKEPKNKTFLRCEAK
NYSGRFTCWWLTTISTDLTFSVKSSRGSSDPQGVTCGAATLSAERVRGDNKEYEY
SVECQEDSACPAAEESLPIEVMVDAVHKLKYENYTSSFFIRDIIKPDPPKNLQLKPLK
NSRQVEVSWEYPDTWSTPHSYFSLTFCVQVQGKSKREKKDRVFTDKTSATVICRK
NASISVRAQDRYYSSSWSEWASVPCSGGGGSGGGGSGGGGSRNLPVATPDPGM
FPCLHHSQNLLRAVSNMLQKARQTLEFYPCTSEEIDHEDITKDKTSTVEACLPLELT
KNESCLNSRETSFITNGSCLASRKTSFMMALCLSSIYEDLKMYQVEFKTMNAKLLM
DPKRQIFLDQNMLAVIDELMQALNFNSETVPQKSSLEEPDFYKTKIKLCILLHAFRIRA
VTIDRVMSYLNASHHHHHH*

Human IL-12 (as a payload for CAR-T):

IWELKKDVYVVELDWYPDAPGEMVVLTCDTPEEDGITWTLDQSSEVLGSGKTLTIQ
VKEFGDAGQYTCHKGGEVLSHSLLLLHKKEDGIWSTDILKDQKEPKNKTFLRCEAK
NYSGRFTCWWLTTISTDLTFSVKSSRGSSDPQGVTCGAATLSAERVRGDNKEYEY
SVECQEDSACPAAEESLPIEVMVDAVHKLKYENYTSSFFIRDIIKPDPPKNLQLKPLK
NSRQVEVSWEYPDTWSTPHSYFSLTFCVQVQGKSKREKKDRVFTDKTSATVICRK
NASISVRAQDRYYSSSWSEWASVPCSGGGGSGGGGSGGGGSRNLPVATPDPGM
FPCLHHSQNLLRAVSNMLQKARQTLEFYPCTSEEIDHEDITKDKTSTVEACLPLELT
KNESCLNSRETSFITNGSCLASRKTSFMMALCLSSIYEDLKMYQVEFKTMNAKLLM
DPKRQIFLDQNMLAVIDELMQALNFNSETVPQKSSLEEPDFYKTKIKLCILLHAFRIRA
VTIDRVMSYLNAS*

CBD-human IL-12 (for production in HEK293F):

CSQPLDVILLLDGSSSFPASYFDEMKSFAKAFISKANIGPRLTQVSVLQYGSITTIDVP
WNVVPEKAHLLSLVDVMQREGGPSQIGDALGFAVRYLTSEMHGARPGASKAVVILV
TDVSVDSVDAAADAARSNRVTVFPIGIGDRYDAAQLRILAGPAGDSNVVKLQRIEDL
PTMVTLGNSFLHKLCSGFVRIGGGGSGGGSIWELKKDVYVVELDWYPDAPGEMVVL
TCDTPEEDGITWTLDQSSEVLGSGKTLTIQVKEFGDAGQYTCHKGGEVLSHSLLLL
HKKEDGIWSTDILKDQKEPKNKTFLRCEAKNYSGRFTCWWLTTISTDLTFSVKSSR
GSSDPQGVTCGAATLSAERVRGDNKEYEYSVECQEDSACPAAEESLPIEVMVDAV
HKLKYENYTSSFFIRDIIKPDPPKNLQLKPLKNSRQVEVSWEYPDTWSTPHSYFSLT
FCVQVQGKSKREKKDRVFTDKTSATVICRKNASISVRAQDRYYSSSWSEWASVPC

SGGGGSGGGGSGGGGSRNLPVATPDPGMFPCLHHSQNLLRAVSNMLQKARQTL
EFYPCTSEEIDHEDITKDKTSTVEACLPLELTKNESCLNSRETSFITNGSCLASRKTS
FMMALCLSSIYEDLKMYQVEFKTMNAKLLMDPKRQIFLDQNMLAVIDELMQALNFN
SETVPQKSSLEEPDFYKTKIKLCILLHAFRIRAVTIDRVMSYLNASHHHHHH*

CBD-human IL-12 (as a payload for CAR-T):

CSQPLDVILLLDGSSSFPASYFDEMKSFAKAFISKANIGPRLTQVSVLQYGSITTIDVP
WNVVPEKAHLLSLVDVMQREGGPSQIGDALGFAVRYLTSEMHGARPGASKAVVILV
TDVSVDSVDAAADAARSNRVTVFPIGIGDRYDAAQLRILAGPAGDSNVVKLQRIEDL
PTMVTLGNSFLHKLCSGFVRIGGGSGGGSIWELKKDVYVVELDWYPDAPGEMVVL
TCDTPEEDGITWTLDQSSEVLGSGKTLTIQVKEFGDAGQYTCHKGGEVLSHSLLLL
HKKEDGIWSTDILKDQKEPKNKTFLRCEAKNYSGRFTCWWLTTISTDLTFSVKSSR
GSSDPQGVTCGAATLSAERVRGDNKEYEYSVECQEDSACPAAEESLPIEVMVDAV
HKLKYENYTSSFFIRDIIKPDPPKNLQLKPLKNSRQVEVSWEYPDTWSTPHSYFSLT
FCVQVQGKSKREKKDRVFTDKTSATVICRKNASISVRAQDRYYSSSWSEWASVPC
SGGGGSGGGGSGGGGSRNLPVATPDPGMFPCLHHSQNLLRAVSNMLQKARQTL
EFYPCTSEEIDHEDITKDKTSTVEACLPLELTKNESCLNSRETSFITNGSCLASRKTS
FMMALCLSSIYEDLKMYQVEFKTMNAKLLMDPKRQIFLDQNMLAVIDELMQALNFN
SETVPQKSSLEEPDFYKTKIKLCILLHAFRIRAVTIDRVMSYLNAS*
