# Supplementary material for: Collagen-binding IL-12-armoured STEAP1 CAR-T cells reduce toxicity and treat prostate cancer in mouse models
Source: Nat Biomed Eng. 2025 Oct 23;10(4):630–46. doi: 10.1038/s41551-025-01508-3 (PMC13099423; doi:10.1038/s41551-025-01508-3)
Supplement: Supplementary file 2 — Reporting Summary [file 41551_2025_1508_MOESM2_ESM.pdf]

nature portfolio

Corresponding author(s): John K. Lee, Jun Ishihara

Last updated by author(s): 26th June, 2025

Reporting Summary

Nature Portfolio wishes to improve the reproducibility of the work that we publish. This form provides structure for consistency and transparency in reporting. For further information on Nature Portfolio policies, see our Editorial Policies and the Editorial Policy Checklist.

Statistics

For all statistical analyses, confirm that the following items are present in the figure legend, table legend, main text, or Methods section.

n/a	Confirmed	
☐	☒	The exact sample size (*n*) for each experimental group/condition, given as a discrete number and unit of measurement
☐	☒	A statement on whether measurements were taken from distinct samples or whether the same sample was measured repeatedly
☐	☒	The statistical test(s) used AND whether they are one- or two-sided<br>*Only common tests should be described solely by name; describe more complex techniques in the Methods section.*
☒	☐	A description of all covariates tested
☐	☒	A description of any assumptions or corrections, such as tests of normality and adjustment for multiple comparisons
☐	☒	A full description of the statistical parameters including central tendency (e.g. means) or other basic estimates (e.g. regression coefficient) AND variation (e.g. standard deviation) or associated estimates of uncertainty (e.g. confidence intervals)
☐	☒	For null hypothesis testing, the test statistic (e.g. *F*, *t*, *r*) with confidence intervals, effect sizes, degrees of freedom and *P* value noted<br>*Give P values as exact values whenever suitable.*
☒	☐	For Bayesian analysis, information on the choice of priors and Markov chain Monte Carlo settings
☒	☐	For hierarchical and complex designs, identification of the appropriate level for tests and full reporting of outcomes
☐	☒	Estimates of effect sizes (e.g. Cohen's *d*, Pearson's *r*), indicating how they were calculated

Our web collection on statistics for biologists contains articles on many of the points above.

Software and code

Policy information about availability of computer code

Data collection	BD FACSDiva (9.0.1) was used for flow cytometry and cell sorting.
Data analysis	Data and statistical analyses were performed using Microsoft Excel v16.88 and GraphPad Prism v10.3.0. Flow cytometry data was analysed by using FlowJo (v10.10).<br><br>Transcriptome data analysis was performed using NanoString Spatial Data Analysis software (GeoMx® DSP Software Version 3.0.0.109). Raw files were analyzed for quality control, followed by sequence alignment. Count matrix files were used to perform differential gene expression analysis using DESeq2. For PCA analysis, Fragments Per Kilobase of transcript per Million mapped reads (FPKM) values were normalized by log2+1 transformation and PCA was plotted based on a correlation matrix using the prcomp package v3.6.2. PCA plots were visualized using the factoextra package v1.0.7 and ggpubr package v0.6.0. Pathway analysis was performed using Gene Set Enrichment Analysis. All computational analyses were carried out in RStudio v4.1.0. Heatmaps were generated using the package pheatmap v1.0.12.<br><br>QuPath 0.5.1 was used to quantify CD3+ area in the IHC images. Average channels were used for tissue detection followed by DAB channel for detection of CD3+ stained area.

For manuscripts utilizing custom algorithms or software that are central to the research but not yet described in published literature, software must be made available to editors and reviewers. We strongly encourage code deposition in a community repository (e.g. GitHub). See the Nature Portfolio guidelines for submitting code & software for further information.

Data

Policy information about availability of data

All manuscripts must include a data availability statement. This statement should provide the following information, where applicable:

- Accession codes, unique identifiers, or web links for publicly available datasets
- A description of any restrictions on data availability
- For clinical datasets or third party data, please ensure that the statement adheres to our policy

The main data supporting the results in this study are available within the paper and its Supplementary Information. Source data are provided with this paper. Spatial transcriptomics data are available at the NCBI GEO repository under accession No. GSE300750.

Research involving human participants, their data, or biological material

Policy information about studies with human participants or human data. See also policy information about sex, gender (identity/presentation), and sexual orientation and race, ethnicity and racism.

Reporting on sex and gender	N/A
Reporting on race, ethnicity, or other socially relevant groupings	N/A
Population characteristics	N/A
Recruitment	N/A
Ethics oversight	N/A

Note that full information on the approval of the study protocol must also be provided in the manuscript.

Field-specific reporting

Please select the one below that is the best fit for your research. If you are not sure, read the appropriate sections before making your selection.

☒ Life sciences ☐ Behavioural & social sciences ☐ Ecological, evolutionary & environmental sciences

For a reference copy of the document with all sections, see nature.com/documents/nr-reporting-summary-flat.pdf

Life sciences study design

All studies must disclose on these points even when the disclosure is negative.

Sample size	Sample size was predetermined from pilot experiments and/or experiments that have been done in the past, to obtain statistically significant data (V. Bhatia et al., Nat. Commun. 2023 Apr 11;14(1):2041. doi: 10.1038/s41467-023-37874-2.)
Data exclusions	One tumor section was excluded in NanoString GeoMx DSP WTA because the sectioned sample was heavily damaged. S1-12 (one of the regions of interest in Fig. 6) was excluded in GSEA based on principle component analysis (PCA). For PCA analysis, FPKM values were normalized by log2+1 transformation and PCA was plotted based on a correlation matrix using the prcomp package v3.6.2.
Replication	Experiments have been repeated multiple times using different independent biological samples with similar experimental conditions or otherwise mentioned in the figure legends, main text or methods. Replicates were reproducible.
Randomization	Mice were purchased from the below indicated vendors, numbered and randomized before treatment.
Blinding	Histological analyses were performed blindly. For tumor measurements, experimenters were not blinded (as it is commonly accepted in the field).

Reporting for specific materials, systems and methods

We require information from authors about some types of materials, experimental systems and methods used in many studies. Here, indicate whether each material, system or method listed is relevant to your study. If you are not sure if a list item applies to your research, read the appropriate section before selecting a response.

Materials & experimental systems

n/a	Involved in the study
☐	☒ Antibodies
☐	☒ Eukaryotic cell lines
☒	☐ Palaeontology and archaeology
☐	☒ Animals and other organisms
☒	☐ Clinical data
☒	☐ Dual use research of concern
☒	☐ Plants

Methods

n/a	Involved in the study
☒	☐ ChIP-seq
☐	☒ Flow cytometry
☒	☐ MRI-based neuroimaging

Antibodies

Antibodies used

Flow cytometry:
anti-human STEAP1 (Vandortuzumab, Invitrogen)
Alexa Fluor 594 AffiniPure F(ab')2 Fragment Donkey anti-human IgG (H+L) (Jackson ImmunoResearch, 709-586-149)
Biotin-SP-conjugated AffiniPure F(ab')2 Fragment Goat Anti-Human IgG, F(ab')2 Fragment Specific (Jackson ImmunoResearch 109-066-006)
anti-pSTAT4 Alexa Fluor 647 (pY693, BD)
anti-mouse CD45.2 APC-Cy7 (30-F11, Biolegend)
anti-mouse CD3 BUV395 (145-2C11, BD)
Anti-mouse CD8 BV510 (53-6.7, Biolegend)
anti-mouse NK1.1 PerCP-Cy5.5 (PK136, Biolegend)
anti-mouse CD4 BUV805 (GK1.5, BD)
anti-mouse CD8 Alexa Fluor 700 (53-6.7, Biolegend)
anti-mouse PD-1 BV605 (29F.1A12, Biolegend)
anti-mouse CTLA-4 APC (UC10-4B9, Biolegend)
anti-mouse CD11b APC-Cy7 (M1/70, Invitrogen)
anti-mouse Ly6G BUV737 (1A8, BD)
anti-mouse Ly6C Alexa Fluor 488 (HK1.4, Biolegend)
anti-mouse CD19 BV785 (6D5, Biolegend)
anti-mouse CD11c PE-Cy7 (HL3, BD)
anti-mouse MHC-II (I-A/I-E) BV711 (M5/114.15.2, Biolegend)
anti-mouse CD103 BV605 (2E7, Biolegend).
anti-mouse CD16/32 (93, Biolegend)
anti-mouse IL-12 PE (C15.6, BD)
anti-human CD8 FITC (SK1, Biolegend)
anti-human IFN-γ APC(4S.B3, Biolegend)
anti-human IL-12 PE (20C2, 1:100, BD)
PE-conjugated Protein L (Sino Biological, 11044-H07E-P)
Biotinylated recombinant protein L (Thermo Scientific)

Immunohistochemistry:
rabbit anti-CD3 antibody (Thermo Fisher, MA5-14524)
PowerVision Poly-HRP anti-rabbit IgG (Leica Biosystems)

ELISA
Peroxidase AffiniPure F(ab')$_2$ Fragment Goat Anti-Mouse IgG (H+L) (Jackson ImmunoResearch, 115-036-003)
mouse IFN-γ ELISA kit (Invitrogen, 88-7314-77)
Human IL-12 p70 DuoSet ELISA (R&D Systems, DY1270-05)

Cell culture:
anti-mouse IL-12 p40 (C17.8, Bio X Cell)
Biotin-SP (long spacer) AffiniPure F(ab')2 Fragment Donkey Anti-Human IgG (H+L) (Jackson ImmunoResearch, 709-066-149)
anti-human CD3 (clone OKT3, Miltenyi Biotec)

Animal experiments
anti-CTLA-4 (9H10, Bio X cell)
anti-PD-1 (RMP1-14, Bio X cell)

Validation

All antibodies were purchased from the vendors mentioned above. Commercial antibodies have been validated by manufacturers based on specific assays, such as side-by-side comparison with an isotype control antibody on flow cytometry, western blot comparin cell lines with or without target expression, and staining of multiple tissues with a known expression pattern of target proteins. Detailed validation information for each antibody is available on datasheet through the manufacturer's website.

Eukaryotic cell lines

Policy information about cell lines and Sex and Gender in Research

Cell line source(s)

HEK293T (CRL-3216), RM9 (CRL-3312), MyC-CaP (CRL-3255), 22Rv1 (CRL-2505) and Jurkat (TIB-152) cells were obtained from

Cell line source(s)	ATCC. HEK293F (Discontinued. Parental cell line of freestyle 293) was obtained from Thermo Fisher Scientific. PLAT-E (RV-101) was obtained from Cell Biolabs.
Authentication	Cell line authentication was done via short tandem repeat (STR) profiling at the IDEXX BioAnalytics, 4011 Discovery Drive, Columbia, MO 65201. Jurkat cells were not authenticated.
Mycoplasma contamination	All cell lines routinely tested negative for Mycoplasma contamination.
Commonly misidentified lines (See ICLAC register)	None.

Animals and other research organisms

Policy information about studies involving animals; ARRIVE guidelines recommended for reporting animal research, and Sex and Gender in Research

Laboratory animals	6 to 9 weeks old male C57BL/6J mice and FVB/N mice, 3 to 5 weeks old male NSG mice were obtained from Charles River UK. For animal experiment at UCLA, male NSG mice (4 to 6 weeks) were obtained from The Jackson Laboratory.
Wild animals	No wild animals were used in this study.
Reporting on sex	Male mice were used to study prostate cancer.
Field-collected samples	No field-collected samples were used in this study.
Ethics oversight	All the animals used in Imperial College London were handled in accordance with the 1986 Animal Scientific Procedures Act and under a United Kingdom Government Home Office-approved project license and overseen by ethical committees of Imperial College London. All mouse studies performed in UCLA were in accordance with protocols approved by the UCLA Institutional Animal Care and Use Committee, known as the Chancellor's Animal Research Committee (ARC).

Note that full information on the approval of the study protocol must also be provided in the manuscript.

Plants

Seed stocks	N/A
Novel plant genotypes	N/A
Authentication	N/A

Flow Cytometry

Plots

Confirm that:

☒ The axis labels state the marker and fluorochrome used (e.g. CD4-FITC).

☒ The axis scales are clearly visible. Include numbers along axes only for bottom left plot of group (a 'group' is an analysis of identical markers).

☒ All plots are contour plots with outliers or pseudocolor plots.

☒ A numerical value for number of cells or percentage (with statistics) is provided.

Methodology

Sample preparation	For phenotyping of in vitro cultured cells, cells were either resuspended in cold PBS (-) and stained with BD Horizon Fixable Viability Stain 510 (BD) or Live/Dead Aqua (Thermo Fisher) before antibody staining, or directly stained with specific antibodies.<br><br>For pSTAT4 assay, T cells were stimulated with the indicated concentrations of IL-12 variants at 37°C for 15 min. Cells were fixed with BD Phosflow Lyse/Fix buffer and permeabilized with BD Phosflow Perm Buffer III according to manufacturer's instructions. Cells were stained with anti-pSTAT4 Alexa Fluor 647 (pY693, BD) and Anti-mouse CD8α BV510 (53-6.7, Biolegend).<br><br>For immune infiltrates analysis using RM9-hSTEAP1 tumors, tumors were collected and cut into small pieces using surgical

	scissors and digested in DMEM supplemented with 2% FBS, 2 mg/mL collagenase D (Sigma-Aldrich) and 40 µg/mL DNase I (Roche) for 30 min at 37°C. Single-cell suspensions in DMEM supplemented with 2%FBS were prepared from digested tumors using a 70 µm cell strainer (Thermo Fisher Scientific). Red blood cells were lysed with ACK lysing buffer (Gibco) for 5 minutes at room temperature and neutralized with PBS (-). Cells were stained with BD Horizon Fixable Viability Stain 510 (BD). Fc receptors were blocked using purified anti-mouse CD16/32 antibody (93, Biolegend). Cells were stained with a cocktail of anti-mouse antibodies and fixed with eBioscience IC Fixation Buffer (Invitrogen).
Instrument	FACSymphony S6 (primary human CAR-T sorting)<br>FACSymphony A5 (Fig. 8A, B)<br>FACSymphony A3 (All the other data).
Software	Data collection: FACSDiva (BD)<br>Analysis: FlowJo v10.10.0. (BD)
Cell population abundance	More than 80% of sorted and expanded primary human T cells used in in vitro functional assays were hSTEAP1 CAR+, confirmed by staining with biotin-anti-Fab (Jackson Immunoreseaerch, 109-066-006) followed by PE-streptavidin (ThermoFisher Scientific). Unsorted CAR-T cell abundance was assessed by flow cytometry a day before or on the day of experiments.
Gating strategy	The gating strategies used were described in supplementary figures 5 and 6.

☒ Tick this box to confirm that a figure exemplifying the gating strategy is provided in the Supplementary Information.
